# Transforming growth factor-β1 protects mechanically injured cortical murine neurons by reducing trauma-induced autophagy and apoptosis

**DOI:** 10.3389/fncel.2024.1381279

**Published:** 2024-05-28

**Authors:** Yanlei Li, Huixiong Deng, Hengyao Zhang, Lin Yang, Shenmiao Wang, Haoyang Wang, Jiacheng Zhu, Xiaoning Li, Xiaoxuan Chen, Yinhong Lin, Rui Li, Gefei Wang, Kangsheng Li

**Affiliations:** ^1^Guangdong Provincial Key Laboratory of Infectious Diseases and Molecular Immunopathology, Shantou University Medical College, Shantou, Guangdong, China; ^2^Department of Radiology, The Second Affiliated Hospital, Medical College of Shantou University, Shantou, Guangdong, China; ^3^Department of Orthopaedics, The Third Xiangya Hospital, Central South University, Changsha, Hunan, China

**Keywords:** trauma-injured, cortical neurons, transforming growth factor-β1, autophagy, apoptosis, lysosomal

## Abstract

Transforming growth factor β1 (TGF-β1) has a neuroprotective function in traumatic brain injury (TBI) through its anti-inflammatory and immunomodulatory properties. However, the precise mechanisms underlying the neuroprotective actions of TGF-β1 on the cortex require further investigation. In this study, we were aimed to investigate the regulatory function of TGF-β1 on neuronal autophagy and apoptosis using an *in vitro* primary cortical neuron trauma-injury model. LDH activity was assayed to measure cell viability, and intracellular [Ca^2+^] was measured using Fluo-4-AM in an *in vitro* primary cortical neuron trauma-injury model. RNA-sequencing (RNAseq), immunofluorescent staining, transmission electron microscopy (TEM), western blot and CTSD activity detection were employed. We observed significant enrichment of DEGs related to autophagy, apoptosis, and the lysosome pathway in trauma-injured cortical neurons. TEM confirmed the presence of autophagosomes as well as autophagolysosomes. Western blot revealed upregulation of autophagy-related protein light chain 3 (LC3-II/LC3-I), sequestosome 1 (SQSTM1/p62), along with apoptosis-related protein cleaved-caspase 3 in trauma-injured primary cortical neurons. Furthermore, trauma-injured cortical neurons showed an upregulation of lysosomal marker protein (LAMP1) and lysosomal enzyme mature cathepsin D (mCTSD), but a decrease in the activity of CTSD enzyme. These results indicated that apoptosis was up-regulated in trauma- injured cortical neurons at 24 h, accompanied by lysosomal dysfunction and impaired autophagic flux. Notably, TGF-β1 significantly reversed these changes. Our results suggested that TGF-β1 exerted neuroprotective effects on trauma- injured cortical neurons by reducing lysosomal dysfunction, decreasing the accumulation of autophagosomes and autophagolysosomes, and enhancing autophagic flux.

## 1 Introduction

Traumatic brain injury (TBI) is a significant global public health and economic concern, leading to substantial mortality and morbidity among young individuals (Rosenfeld et al., 2012; Andelic, 2013). The primary injury directly causes brain trauma, ultimately resulting in neuronal cell death (Shafi et al., 2014; Hutchinson et al., 2016; Timmons et al., 2019). Mechanisms such as the pro-inflammatory response, oxidative stress, local hypoxia/reoxygenation, as well as accumulation of neurotoxic substances contribute to damage and degradation in the surrounding microenvironment, leading to persistent neurodegeneration (Ji et al., 2012; Chao et al., 2018). Over time, secondary damage develops through the activation of apoptosis, necroptosis, pyroptosis, autophagy, and other necrotic inflammatory programs, resulting in neuronal death and pathological and functional changes in the brain (Ito et al., 2015; Arrázola et al., 2019; Chao et al., 2019). Despite increased understanding of the biological mechanisms involved in neurorepair after TBI, effective therapeutic drugs and methods for neuroprotection remain limited, primarily due to the heterogeneity of pathology as well as the complexity of secondary injury mechanisms in TBI (Kalra et al., 2022).

Among the complex cytokine networks induced after TBI, transforming growth factor-β1 (TGF-β1) is crucial as an anti-inflammatory and neuroprotective mediator. Research has shown that TGF-β1 has the potential to decrease neuroinflammation and prevent further damage following the initial injury. It acts as the main effector cytokine and may serve as a neuroprotective agent in neurodegenerative disorders, aiding in the management of neuronal damage in conditions like Parkinson’s/Alzheimer’s diseases (Goodman et al., 1990; Ott et al., 1994; Bell et al., 1997; Kossmann et al., 1997; Morganti-Kossmann et al., 1999). Importantly, TGF-β1 treatment has been found not only to participate in immunomodulation and exert neuroprotective effects but also to promote the recovery of injured neurons and reduce axonal damage within 1 day after TBI. In a previous study, our team discovered that the inhibitory effect of TGF-β1 on the L-type Ca^2+^ channels (LTCCs) mediated elevation of intracellular Ca^2+^ concentration among mouse cortical neurons stimulated by KCl. It was achieved through the activation of the c-Jun N-terminal kinases (JNK1/2), and p38 mitogen-activated protein (MAP) kinases (MAPK), MAPK/extracellular signal-regulated kinase (ERK) kinase (MEK) pathways (Liu et al., 2018). Additionally, we observed that TGF-β1 stimulates the p38 MAPK pathway to upregulate the C_av_1.2 channel, thereby enhancing cortical neuron activity in trauma-injured mice and inhibiting cell apoptosis (Li et al., 2022). In summary, TGF-β1 regulates neuronal regeneration by modulating immune responses, cellular activity, scar formation, and neurite growth (Morganti-Kossmann et al., 1999). However, the precise mechanisms underlying the neuroprotective effects of TGF-β1 on mechanically damaged neurons are not fully understood. To address this gap, we employed an *in vitro* model of primary trauma-injured cortical neurons to investigate the impact of TGF-β1 on cortical neurons subjected to trauma injury. This study utilized RNA sequencing (RNA-seq) technique to reveal crucial pathways and hub genes associated with the downstream effects of TGF-β1. We observed that mechanically impaired neurons exhibited autophagy dysfunction, which could be alleviated by treatment with TGF-β1 (10 ng/mL), leading to neuroprotective effects. Additionally, our experimental results indicated that the TGF-β1-controlled autophagy pathway helped to mitigate the upregulation of autophagy, which in turn prevented dysfunctional lysosomal storage, enhances autophagic flux, and inhibits apoptosis, ultimately playing a neuroprotective role. Therefore, this investigation was aimed to enhance our understanding of the protective mechanism of TGF-β1 on cortical neurons after injury, providing additional therapeutic targets for TBI.

## 2 Materials and methods

### 2.1 Animals and cells

C57BL/6 J mice were obtained from Beijing Vital Liver Laboratory Animal Technology Co., LTD. (Beijing, China). All experiments followed the guidelines of the National Institutes of Health Guide for the Care and Use of Laboratory Animals and were approved by the Ethical Standards for Animal Care and Use of Research at Shantou University Medical School (approval grant no SUMC2022-413). Pregnant C57BL/6 J mice, aged between 12 and 18 weeks, were housed in a controlled environment with a temperature of 21 ± 2°C, humidity ranging from 30 to 70%, and a 12-h light/dark cycle. They had *ad libitum* access to food and water. Within 24 h of birth, neonatal C57BL/6 J mice were anesthetized using isoflurane (R510-22, RWD Life Science, China) at an induction concentration of 5% and maintenance concentration of 2.5%. Brain tissue was extracted, and cortical neurons were isolated. The cerebral cortices, excluding the hippocampus and dura mater, were treated with 2 mL of papain (P3125, Sigma, United States) diluted to 60 U/mL at 37°C for 20 min to obtain cells. Subsequently, 1 × 10^6^ cells were cultured on poly-D-lysine-treated glass coverslips (E607014-0002, Industrial Biotech, China) in 35 mm cell culture dishes/plates, supplemented with 2 mL of neurobasal medium in a 5% CO_2_ / 95% air atmosphere.100 Gluamax™-1 (2,003,971, Gibco, United States) and SM1 (05711, stem cell, United States) were added to 2% of the neurobasal medium (05790, stem cell, United States) immediately before use. Half of the medium was changed every 3 days. On day 1, arabinosylcytosine (5 μmol, 147–94-4, MERCK, United States), a selective inhibitor of DNA synthesis, was added to the culture and incubated for 24 h to minimize glial contamination. All experiments were conducted between day 8 and day 10.

### 2.2 Cell viability assay with LDH

Cortical neurons were cultured in 24-well plates for 7 days. The medium was then replaced, and the neurons were scratched and treated with TGF-β1 (10 ng/mL). After 24 h, the supernatant was aspirated, and the released lactate dehydrogenase (LDH) was measured using the LDH Activity Assay Kit (Beyotime, China) according to the manufacturer’s instructions, and served as a marker of cell death in the culture medium.

### 2.3 *In-vitro* model of trauma-injured cortical neurons

A model of trauma-injured cortical neurons was established by inducing mechanical injury on primary cortical neurons *in vitro* (Katano et al., 1999). Parallel scratches were created on the cultured cell plates (12 × 12 scratches in six-well plates) using a sterile 21-gauge needle (0.6 mm, HD, China). The plates were then incubated for 24 h.

### 2.4 Western blotting analysis

Cultured cortical neurons were disrupted using ristocetin-induced platelet aggregation (RIPA) buffer (P10013B, Beyotime, China) containing phenylmethylsulfonyl fluoride (PMSF) (ST506, Beyotime, China) and Halt™ Phosphatase Inhibitor Cocktail (78,427, Thermo Fisher Scientific, United States). Protein samples were separated on a 10% sodium dodecyl sulfate polyacrylamide gel electrophoresis (SDS-PAGE) gel in Glycine-Tris buffer and transferred onto polyvinylidene fluoride (PVDF) membranes (09609A, Life Sciences, United States). The membrane was blocked with 5% non-fat milk (Anchor, New Zealand) in TBST buffer at room temperature for 1 h, followed by overnight incubation at 4°C with primary antibodies for LC-3B (AB192890, anti-rabbit, 1:2000, abcam, United States), β-actin (A1978, anti-mouse, 1:5000, Sigma, United States) as the internal control, Cleaved-Caspase3 (AB214430, anti-rabbit, 1:5000, abcam, United States), lysosomal marker protein (LAMP1) (AB202843, anti-rabbit, 1:1000, abcam, United States), Anti- sequestosome 1 (SQSTM1)/P62 antibody (AB109012, anti-rabbit, 1:1000, abcam, United States), and Cathepsin D Polyclonal antibody (21327-1-AP, anti-rabbit, 1,5,000, Proteintech, China). After washing with Tris-buffered saline with Tween (TBS/Tween), PVDF membranes were incubated with horseradish peroxidase (HRP)-conjugated secondary antibody (Goat anti-Rabbit, 1:5000, Sigma, United States) and washed again with TBS/Tween. Protein bands were detected using an Enhanced Chemiluminescence (ECL) detection reagent (HYK1005, Med Chem Express, United States). The optical density of protein bands was quantified using Quantity One software from Bio-Rad (Hercules, CA, United States).

### 2.5 Real-time quantitative polymerase chain reaction (RT-PCR)

The total RNA from the primary cortical neurons was extracted using TRIzol reagent (15,596,026, Thermo Fisher Scientific, United States). Subsequently, the RNA was reverse transcribed into cDNA using a reverse transcription kit (R333-01, Vazyme, China) and the Power-up ™ SYBR™ Green master mix (00931764, Thermo Fisher Scientific, United States). The mRNA expression levels of mouse Beclin1, SQSTM1/P62, LAMP1, cathepsin D (CTSD), cathepsin B (CTSB), and β-actin were analyzed using five complementary primers synthesized by Guangzhou IGE Biotechnology Ltd. (IGE, China). It should be noted that β-actin was used as the control, as indicated in Supplementary Table S1. The expression level was determined using the 2^-△△Ct^ method and normalized based on the expression of β-actin.

### 2.6 Measurement of intracellular [Ca^2+^] using Fluo-4-AM

Neurons were incubated with 5 μM Fluo-4-AM at 37°C for 40 min. Afterward, the cells were washed with Hank’s balanced salt solution to remove extracellular Fluo-4-AM. The laser confocal scanning system (LSM 880, Zeiss, Germany) was utilized to detect the fluorescence signal. Fluo-4 was excited at 488 nm using an argon laser, and emission was measured at 530 nm. Changes in intracellular Ca^2+^ concentration were determined by calculating the ratio of fluorescence intensity (ΔF) against its background, represented as ΔF/F0, where F0 denotes the baseline fluorescence averaged from 0 s. F represents the cell fluorescence density at different time points. ΔF = F - F0 indicates the fluctuation in cell fluorescence density at different time points. Neurons were scanned for 1 h for imaging in each test.

### 2.7 TUNEL analysis

The neuronal cells of the cortex were cultured in prior for a duration of 8 days before undergoing traumatic injury and subsequent treatment utilizing TGF-β1 (10 ng/mL). Post 24 h of this treatment regimen, the culture medium was wholly discarded, followed by a double wash of the cells with PBS. Subsequently, these cortical neurons were subjected to a fixation process using 4% paraformaldehyde for an entire half-hour. The detection of cellular apoptosis was undertaken employing the terminal deoxynucleotidyl transferase dUTP nick end-labelling detection kit (C1086, TUNEL, Beyotime, China) in strict accordance with the manufacturer’s delineated guidelines.

### 2.8 Immunofluorescent staining

To examine the distribution of intracellular LC-3B expression in neurons, cortical neurons were cultured in individual dishes and allowed to grow for 7 days before scratching and drug treatment. After 24 h of TGF-β1 treatment, the medium was discarded, and neurons were triply washed with cold phosphate buffered saline (PBS). Then, the neurons were fixed in 4% paraformaldehyde for 5 min, followed by triply washed with PBS. Next, the samples were blocked with 1% bovine serum albumin (BSA) for 1 h. Anti-LC-3B (AB192890, anti-rabbit, use a concentration of 0.1 μg/mL, abcam, United States) and anti- microtubule-associated protein 2 (MAP2) antibodies (M3696, 1:200, sigma, United States) were incubated at 4°C for 2 h. Afterwards, the samples were washed three times with PBS and treated with Alexa Fluor 488-conjugated anti-rabbit IgG (A0428, anti-mouse, 1:500, Beyotime, China) and Alexa Fluor CY3-conjugated goat anti-mouse IgG (A0516, anti-rabbit, 1:500, Beyotime, China) for 1 h. Nucleus DNA was labeled with Hoechst 33258 (C1011, Beyotime, China) for 10 min at room temperature. The samples were then washed three times with PBS, and the plates were sealed using an anti-fluorescent surfactant. Finally, the Confocal 800 imaging system (LSM 800, Zeiss, Germany) was used to capture images.

### 2.9 TEM

Neuron precipitates were collected after centrifugation. TEM fixative (G1102, Servicebio, China) was added to the tube to resuspend the precipitation and fix it at 4°C for preservation and transportation. After discarding the supernatant, 0.1 M PBS (pH 7.4) was added, and the precipitate was resuspended and washed with PBS for 3 min. A 1% agarose solution (A6338, Macklin, China) was prepared by heating and dissolving it in advance. Once cooled, the agarose solution was added to the eppendorf tube. Before the agarose solidified, the precipitate was suspended with forceps and wrapped in the agarose. Agarose blocks containing the samples were post-fixed with 1% OsO4 (18,456, Ted Pella Inc., United States) in 0.1 M PBS (pH 7.4) for 2 h at room temperature. After removing OsO4, the tissues were rinsed in 0.1 M PBS (pH 7.4) three times for 15 min each. Dehydration was carried out at room temperature using a series of ethanol concentrations: 30% ethanol (100,092,183, national pharmaceutical reagent, China) for 20 min, 50% ethanol for 20 min, 70% ethanol for 20 min, 80% ethanol for 20 min, 95% ethanol for 20 min, two changes of 100% ethanol for 20 min each, and finally two changes of acetone for 15 min each. Resin penetration and embedding were conducted as follows: Acetone (10,000,418, National medicine reagent, China): EMBed 812 (90529–77-4, SPI, United States) = 1:1 for 2–4 h at 37°C; Acetone: EMBed 812 = 1:2, 37°C overnight; pure EMBed 812 at 37°C for 5–8 h; pour pure EMBed 812 into the embedding model, insert the tissue into pure EMBed 812, then leave it overnight at 37°C. The resin-embedded model and samples were transferred to a 65°C oven for polymerization for more than 48 h. The resin blocks were then removed from the embedding model and kept at room temperature for future use. The resin blocks were cut into 60–80 nm thin sections using a supermicrotome (Leica UC7, Leica, Germany) and placed on 150 copper grids. The grids were stained with 2% uranium acetate saturated solution to avoid photostaining for 8 min and washed three times with 70% ethanol, followed by three washes with ultrapure water. 2.6% lead citrate was used to avoid CO_2_ staining for 8 min, and then rinsed three times with ultrapure water. After drying with filter paper, the copper grid was placed into a grid plate and dried overnight at room temperature. The copper grids were observed under JEM-F200 TEM (JEOL, Japan), and images were captured.

### 2.10 Detection of CTSD activity

CTSD activity was assessed utilizing a Fluorometric Assay Kit (ab65302, Abcam, United States). The kit utilizes a fluorescence-based method with a specific CTSD substrate sequence (GKPILFFRLK(Dnp)D-R-NH2) labeled with methyl coumarylamide (MCA). Samples were prepared complying with the instructions provided by the manufacturer. Cells (1 × 10^6^) were collected for each condition, washed with ice-cold PBS, and resuspended in 100 μL of PBS. The cells were homogenized by repeated pipetting and then centrifuged at 4°C and 100 × g for 5 min. The supernatant was carefully decanted without disturbing the cell pellet, which was transferred to a new tube containing 200 μL of chilled CD Cell Lysis Buffer, AUTOPHAGY 1539, and incubated on ice for 10 min. After another centrifugation step at 4°C and 21,000 x g for 5 min to remove insoluble material, the cleared cell lysate was transferred to a new tube. 50 μL of cell lysate was combined with 52 μL of reaction mix, consisting of 50 μL of reaction buffer and 2 μL of substrate. This mixture was then aliquoted into wells of a black 96-well plate (CORNING, 3916) and incubated at 37°C for 30 min. The resulting fluorescence from substrate cleavage was measured using a SynergyH1 microplate reader (F-4700, Hitachi LimitedB, Japan) at Ex/Em = 328/460 nm.

### 2.11 RNA-seq

The total RNA extraction from the samples was carried out, and its integrity was evaluated utilizing the Agilent 2,100 bioanalyzer system. Amplified RNA libraries were prepared for each of the six samples following the Illumina protocol. Clustering of the index-coded samples was performed using the AMPure XP system (Beckman Coulter, Becerly, United States). Subsequently, the library preparations were subjected to sequencing, generating paired-end reads with a length of 150 bp (Illumina NovaSeq 6,000, United States). The paired-end clean reads were aligned to the reference genome using Hisat2 (v2.0.5). StringTie (v1.3.3b) was utilized to assemble the mapped reads for individual samples. Feature Counts (v1.5.0-p3) was employed to quantify the number of reads mapped to each gene. The fragments per kilobase of exon per million fragments mapped (FPKM) value of each gene was calculated based on the gene length and the number of mapped reads. Differential gene expression analysis was performed using the DESeq2 R software version 1.20.0. Genes with an adjusted *p*-value (padj) < = 0.05 and |log2(fold change [FC])| > = 0,were considered as differentially expressed genes (DEGs). DEGs were identified based on the available samples. The ratio of mRNA expression between TGF-β1 treated and untreated cells was determined by calculating the mean of the normalized read count values from each group. Any ratio with |log2(fold change [FC])| > = 0 & padj <= 0.05 in the Trauma group in contrast with the control group was considered a DEG. The RNA-seq data has been deposited in the GEO database at the NCBI with the accession number GSE249554. Gene Ontology (GO) enrichment analysis of DEGs was conducted using the clusterProfiler R package. Kyoto Encyclopedia of Genes and Genomes (KEGG)-based pathway analysis was performed using the Cluster Profiler R package and the DAVID database (v6.8, https://david.ncifcrf.gov/) to identify significantly enriched GO terms and KEGG pathways (padj <0.05) among the DEGs. The online Search Tool for the Retrieval of Interacting Genes (STRING) database (v11.0, https://www.string-db.org/) was utilized to generate protein–protein interaction (PPI) networks and perform construction and module analyses of proteins encoded by the DEGs. Interactions with a combined score of at least 0.4 indicated statistically significance. Cytoscape software (v3.8.2) in combination with the Molecular Complex Detection (MCODE) app (v2.0.0) was employed to cluster the network based on topology and identify densely connected regions (Bader and Hogue, 2003). A degree cutoff of 2, a node score cutoff of 0.2, a k-score of 2, and a maximum depth of 100 were used. For the genes associated with autophagy pathway, hub genes among the DEGs were identified using the degree method through the cytoHubba app (v0.1) in Cytoscape. GO annotation and KEGG (Kyoto Encyclopedia of Genes and Genomes) pathway analysis using the DAVID (Database for Annotation, Visualization and Integrated Discovery) database were performed for the genes within these modules.

### 2.12 Statistical analysis

Statistical analysis was conducted using GraphPad Prism 7 software (GraphPad Software Inc., San Diego, CA, United States). Data comparison between two groups was performed using an unpaired t-test. For normally distributed data, a one-way analysis of variance (ANOVA) followed by Fisher’s LSD multiple comparisons was used for normally distributed data. The Brown-Forsythe and Welch ANOVA tests were employed for data with normal distribution but non-homogeneous variance. The nonparametric Kruskal-Wallis rank ANOVA with post-hoc Dunn’s test was used for non-normally distributed data. Results are presented as the mean ± standard error of the mean (SEM) or standard deviation (SD) as specified. A two-tailed *p*-value of less than 0.05 was considered statistically significant.

## 3 Results

### 3.1 TGF-β1 treatment (10 ng/mL, 24 h) significantly increased the neuronal viability and maintenance Ca^2+^ homeostasis of trauma-injured cortical neurons

**Figure 1:**
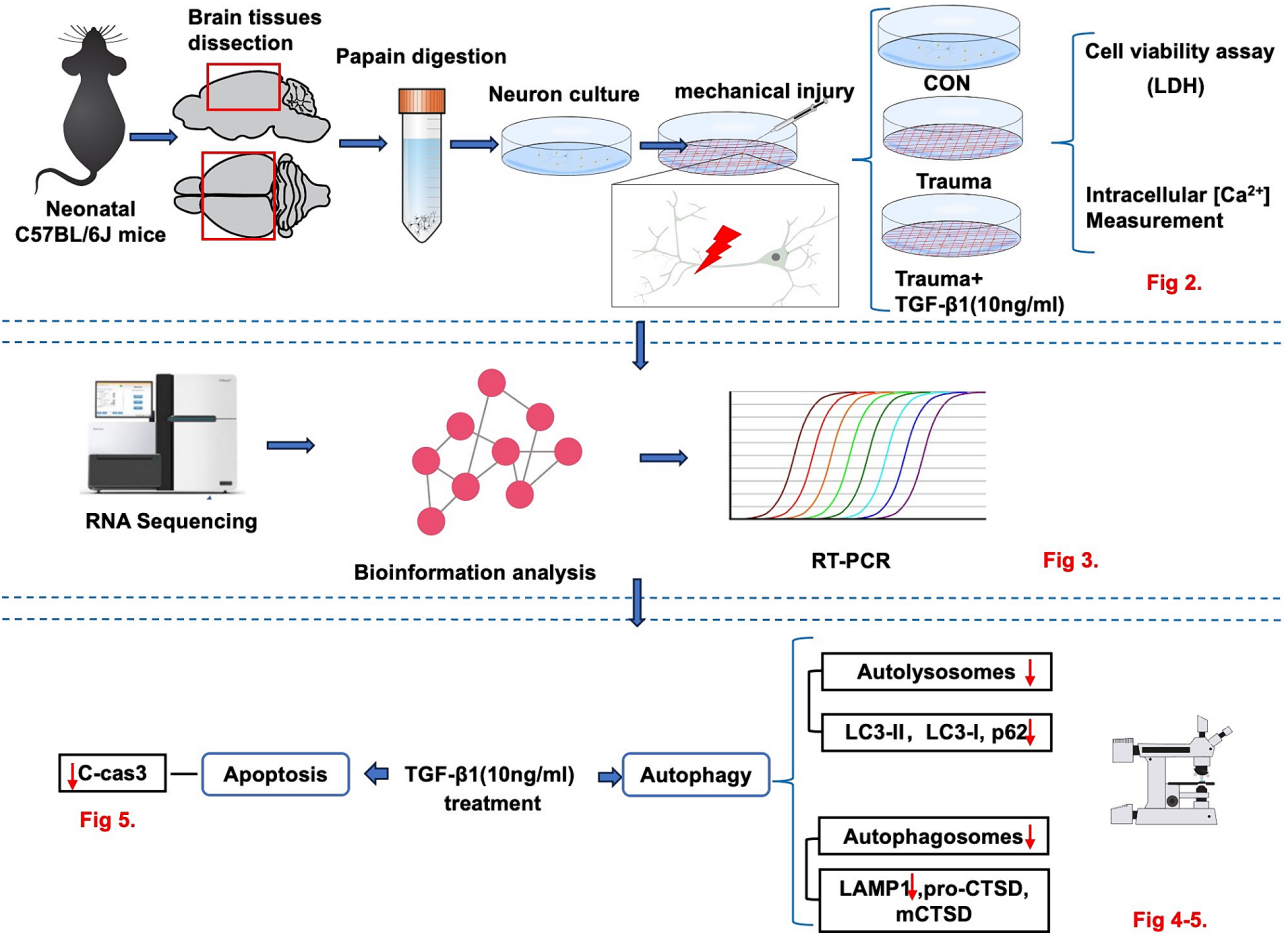
The experimental workflow illustrating the *in vitro* model to investigate the neuroprotective effect of TGF-β1 on trauma-injured cortical neurons by reducing trauma-induced autophagy and apoptosis in murine cortical neurons.

**Figure 2:**
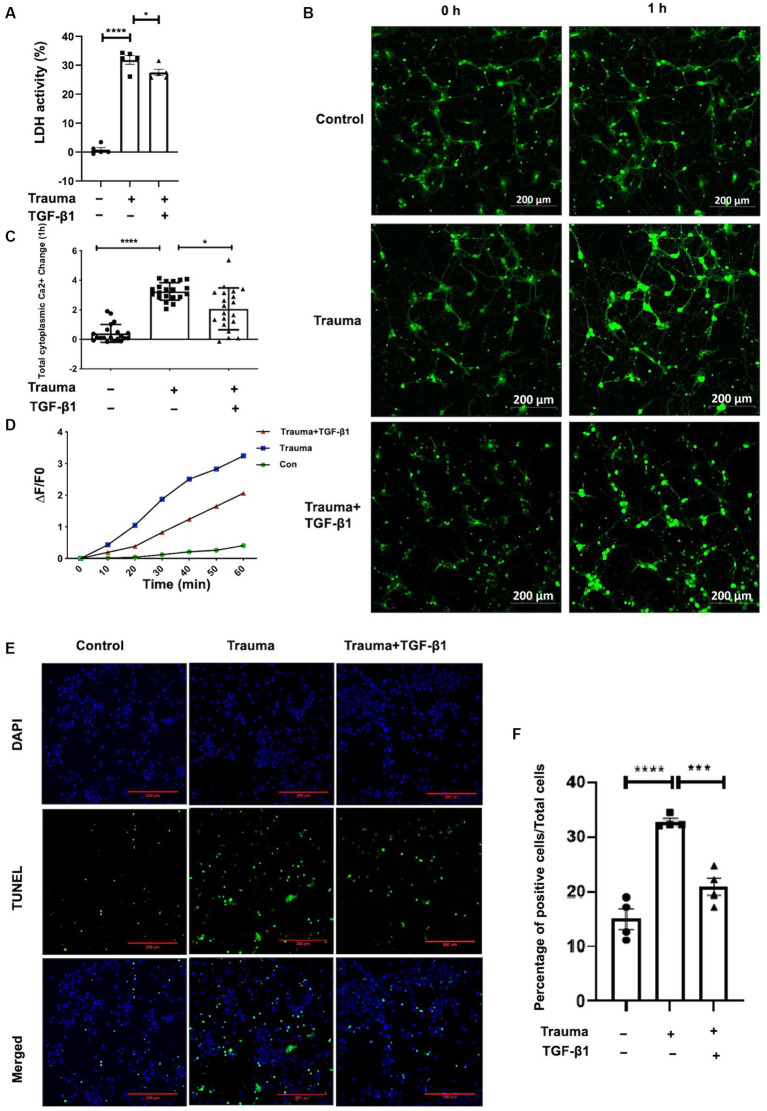
Treatment with TGF-β1 (10 ng/mL) for 24 h significantly increased neuronal viability and maintained Ca^2+^ homeostasis. **(A)** LDH release from trauma-injured cortical neurons of mice after exposure to TGF-β1 (10 ng/mL) for 24 h; **(B)** Representative images of Fluo-4 AM in cortical neurons showing intracellular Ca^2+^ concentration. Scale bar: 200 μm. **(C)** Changes in intracellular Ca^2+^ concentration in TGF-β1-treated trauma-injured neurons at 1 h. **(D)** The temporal trend of intracellular Ca^2+^ concentration in these neurons over 1 h. Each test interval was 10 min. Intracellular Ca^2+^ concentration alterations were calculated by comparing fluorescence intensity (△F) with background, using △F/F0 as an index. F0 represents the baseline fluorescence averaged from 0 s, while F indicates cell fluorescence density at different time points. △F = F-F0 represents changes in cell fluorescence density at different time points. **(E)** TUNEL assay indicating cellular apoptosis; green fluorescence (TUNEL positive cells) and blue staining of the nuclei was Hoechst. **(F)** Neuronal apoptosis with or without trauma injury and TGF-β1 (10 ng/mL) treatment (*n* = 4 for each group). Data are presented as mean ± SD, ^*^p < 0.05, ^**^p < 0.01, ^***^p < 0.001, ^****^p < 0.0001.

Our previous experiments have demonstrated the dose-dependent effect of TGF-β1 on neuronal protection. Treatment with TGF-β1 at a concentration of 10 ng/mL significantly increased neuronal activity and inhibited neuronal apoptosis induced by mechanical injury. However, higher doses of treatment may have a detrimental effect. In this study, we aim to investigate the protective mechanism of TGF-β1 (10 ng/mL) against mechanically damaged cortical neurons, as depicted in Figure 1. To assess neuronal activity, we initially measured LDH discharge in the cellular solution supernatants. As shown in Figure 2A, there was a notable increase in LDH release from the supernatant of the TBI group in contrast with the control group 24 h post-injury. However, after 24 h of treatment with TGF-β1 (10 ng/mL), there was a significant decrease in LDH elevation. These findings indicate that TGF-β1 reduces the cytotoxicity of mechanically injured cortical neurons, thereby preserving cellular activity. Maintaining an appropriate intracellular Ca^2+^ concentration is crucial for normal cortical neuronal function. To quantify alterations intracellular Ca^2+^ concentration after mechanical injury, we employed Fluo-4 AM labeling, observed the neurons using laser confocal fluorescence microscopy, and conducted statistical analysis of fluorescence densities. The data obtained are presented in Figures 2B,C, indicated that mechanically injured neurons experienced a sustained increase intracellular Ca^2+^ concentration, resulting in calcium overload. However, administration of TGF-β1 led to a considerable reduction in the rise of intracellular Ca^2+^ concentration in mechanically injured neurons. Furthermore, TGF-β1 effectively inhibited the continuous rise in intracellular Ca^2+^ concentration caused by mechanical injury, as monitored for 60 min (Figure 2D). These findings suggested that TGF-β1 at a concentration of 10 ng/mL could preserve intracellular Ca^2+^ homeostasis in trauma- injured cortical neurons, thereby exhibiting a neuroprotective effect. The outcomes derived from the TUNEL assay underscored that trauma precipitated a considerably amplified rate of apoptosis, juxtaposed to the control group. Moreover, TGF-β1 (10 ng/mL) remarkably mitigated the rate of apoptosis invoked by trauma (Figures 2E,F).

### 3.2 Alleviation of gene expression levels involved in autophagy and lysosomal pathways within trauma-injured cortical neurons by TGF-β1

**Figure 3:**
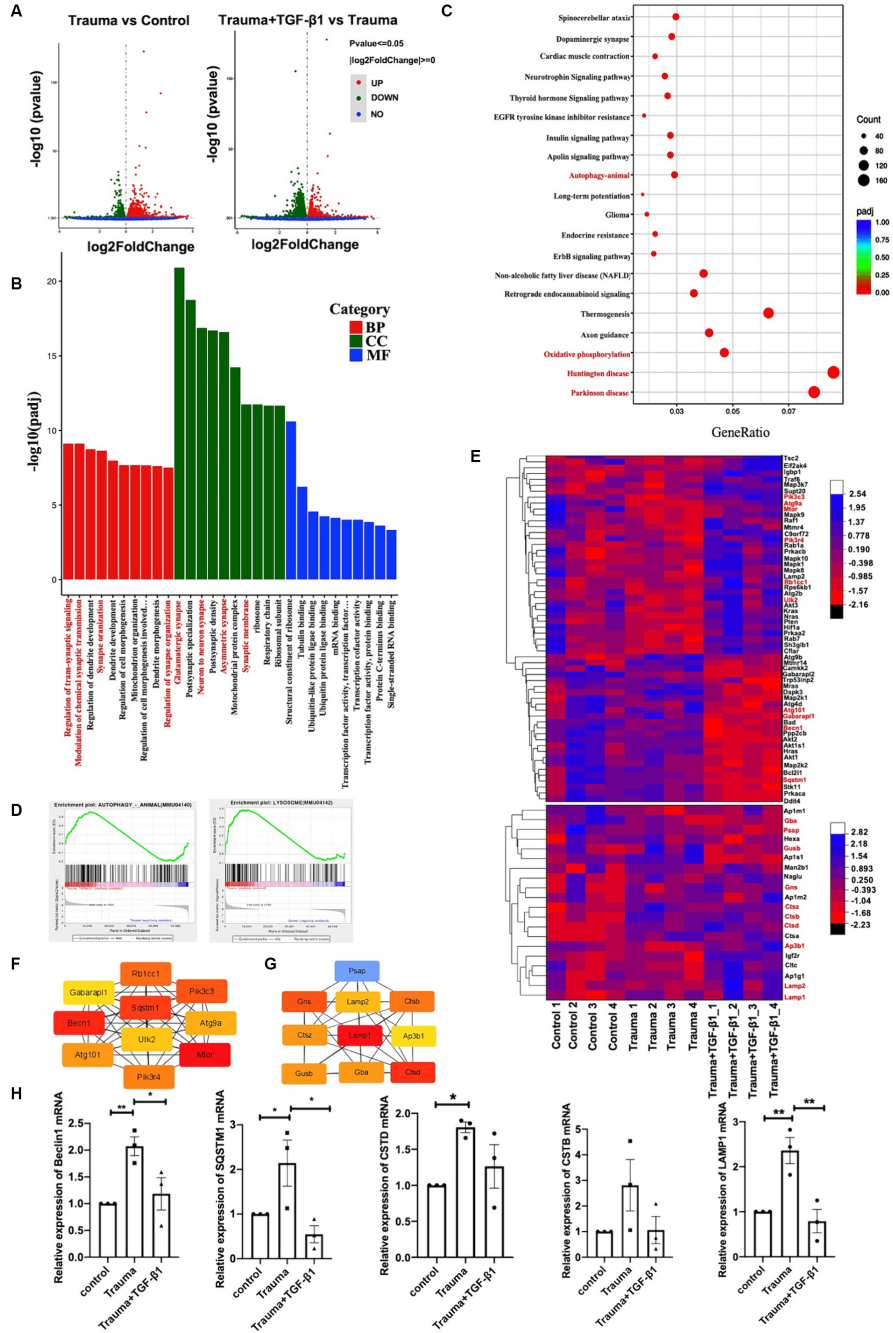
Identification of differentially expressed genes and bioinformatics analysis. **(A)** Volcano plot displaying differentially expressed genes, with upregulated genes (padj <= 0.05 and |log2(fold change [FC])| > = 0) marked in red, and downregulated genes marked in green (padj <= 0.05 and |log2(fold change [FC])| > = 0). ‘padj’ represents adjusted *p* value. **(B)** Top 10 most enriched Gene Ontology (GO) terms of Biological Process (BP), Cellular Component (CC), and Molecular Function (MF), sorted by padj value. GeneRatio represents the ratio of enriched DEGs to total DEGs. **(C)** Trauma *VS* Trauma+TGF-β1 group Top 20 significantly enriched KEGG pathways. GeneRatio represents the ratio of enriched DEGs to total DEGs. Dot size and color represent the number of DEGs enriched in the pathway and its significance, respectively. **(D)** GSEA demonstrating the enrichment of autophagy and lysosomal pathways among differentially expressed genes (DEGs) in the TGF-β1-treated group. **(E)** Autophagy and lysosomal Pathway DEG Enrichment Heat Map showing expression values for the six libraries as normalized FPKM values in the TGF-β1-treated group. Red denotes upregulated DEGs, while blue represents downregulated DEGs. **(F)** Top 10 hub genes of the autophagy pathway within the network ranked using the degree method through cytoHubba in the TGF-β1-treated group. **(G)** Top 10 hub genes of the lysosomal pathway within the network ranked using the degree method through cytoHubba in the TGF-β1-treated group. **(H)** Verification of RNA-seq results using RT-qPCR, ^*^p < 0.05, ^**^p < 0.01, ^***^p < 0.001, ^****^p < 0.0001. Statistical comparisons were performed using one-way ANOVA. Data are presented as mean ± SEM.

To explore the mechanisms underlying the effects of TGF-β1 on trauma-injured cortical neurons, we conducted RNA-seq. We identified 2,696 differentially expressed mRNAs in trauma-injured cortical neurons, with 1,416 upregulated and 1,280 downregulated genes in the TBI group compared to the control group. Furthermore, we observed 4,114 differentially expressed mRNAs between the Trauma and the Trauma +TGF-β1 groups, with 2082 upregulated and 2,332 downregulated genes. The volcano diagram demonstrates significant differences in DEGs between the TGF-β1 treated and control groups (Figure 3A). The transcriptome expression patterns differed between the groups, and TGF-β1 regulated the transcript levels of genes in mechanically injured cortical neurons, including 2,376 unique differential genes (Supplementary Figure S1).To determine the functions / pathways of DEGs, GO, KEGG, as well as Gene Set Enrichment Analysis (GSEA) pathway enrichment analyses were conducted. DEGs upregulated in the trauma group were mainly associated with positive regulation of cell death, cell development regulation, and negative regulation of cell differentiation (Supplementary Figure S2). Conversely, the TGF-β1 treatment group exhibited significant enrichment in various biological processes (BPs)/pathways. Neuronal DEGs were primarily involved in regulating trans-synaptic signaling, modifying chemical synaptic transmission, and regulating dendrite development within BPs. For upregulated DEGs, changes in BP were primarily concentrated in cell migration, protein phosphorylation, phosphorylation, and negative regulation of the apoptotic process. In terms of molecular function (MF), most neuronal DEGs were associated with the structural constituent of ribosomes, with tubulin binding and ubiquitin-like protein ligase binding being the top two abundant subcategories. Regarding cellular components (CC), neuronal DEGs were predominantly linked to glutamatergic synapses, postsynaptic synapses, and neuron-to-neuron synapses (Figure 3B; Supplementary Table S2). The KEGG of trauma-injured cortical neurons with TGF-β1 revealed that most upregulated DEGs were associated with synaptic and dendritic functions in neurons. It also enhanced the Long-term potentiation, the MAPK, apelin, Erbb signaling pathway, as well as the wnt signaling pathway (Supplementary Figure S3A). Most inhibited neuronal DEGs were associated with Parkinson’s disease, Huntington’s disease, thermogenesis (Figure 3C; Supplementary Figure S3B; Supplementary Table S3). Additionally, a GSEA of trauma-injured cortical neurons with TGF-β1 treatment indicated that alterations in cortical neurons were linked to the activation of calcium signaling pathways and apelin signaling pathways. Other pathways affected included ubiquitin-mediated protective catabolism and autophagy, glycolysis/glycogen synthesis, primary immunodeficiencies, propionate metabolism, chemokine signaling pathways, multispecies apoptosis, and acute myeloid leukemia pathways (Supplementary Figure S4).

Through Transcriptional Regulatory Relationships Unraveled by Sentence-based Text mining (TRRUST) database analysis, several transcription factors (TF) were identified among the 4,114 DEGs after TGF-β1 treatment. TFs Smad3 and Hifla were predicted to be significant among the upregulated differential genes in the transcriptome of mechanically injured neurons treated with TGF-β1. Furthermore, the TF SP1 was predicted to be significant among the downregulated DEGs (Supplementary Figure S5). The identified pathways and processes indicate a close relationship between the DEGs and synaptic plasticity. Maintaining protein biosynthesis and degradation balance is crucial for synapses to maintain homeostasis and synaptic plasticity (Bingol and Sheng, 2011). Disruptions in proteostasis can result in functional and ultra-structures deterioration (Douglas and Dillin, 2010). Autophagy plays a critical role in maintaining synaptic homeostasis by turning over synaptic proteins (Hara et al., 2006; Komatsu et al., 2007). Our transcriptome analysis revealed that TGF-β1 suppressed the upregulated autophagy pathway and lysosome-related gene expression in mechanically injured cortical neurons (Figure 3D). Thus, TGF-β1 may exert neuroprotective effects by regulating the autophagy pathway in mechanically injured cortical neurons, helping to maintain synaptic homeostasis.

To further investigate the role of autophagy in this process, we analyzed DEGs related to autophagy and lysosomes. We identified 59 DEGs related to autophagy, with 34 upregulated and 25 downregulated genes (Figure 3E; Supplementary Table S4), as well as 150 DEGs related to lysosomes, with 83 upregulated and 67 downregulated genes (Figure 3E; Supplementary Table S5). Additionally, we performed PPI and hub gene analysis on the 59 autophagy-related genes and 150 DEGs associated with lysosomes (Supplementary Figure S6). Among the top 10 hub genes in the network, ranked by the degree method, were Gabarapl1, Pik3cs, Becn1, SQSTM1, Atg9a, Atg101, UIK2, Rbcc1, Mtor, and Pik3r4, all of which are key molecules in the autophagy pathway (Figure 3F). The top 10 hub genes associated with lysosomes were Psap, Lamp2, Gns, Ctsz, Gusb, LAMP1, Gba, CTSB, Ap3b1, and Ctsd (Figure 3G). To validate the RNA-Seq results, we selected five critical genes in the autophagy pathway (Beclin1, SQSTM/P62, LAMP1, CTSD, and CTSB) for RT-PCR verification. The RT-qPCR findings validated that the expression profiles of these five genes most corresponded to the results from RNA-Seq, providing reliability to the RNA-Seq outcomes (Figure 3H). In summary, evidence from transcriptomic and RT-PCR analyses suggests that autophagy is hindered during mechanical injury of neurons, resulting in neuronal dysfunction and neurodegeneration.

### 3.3 TGF-β1 treatment reduced the count of both autophagosomes and autophagic lysosomes

**Figure 4:**
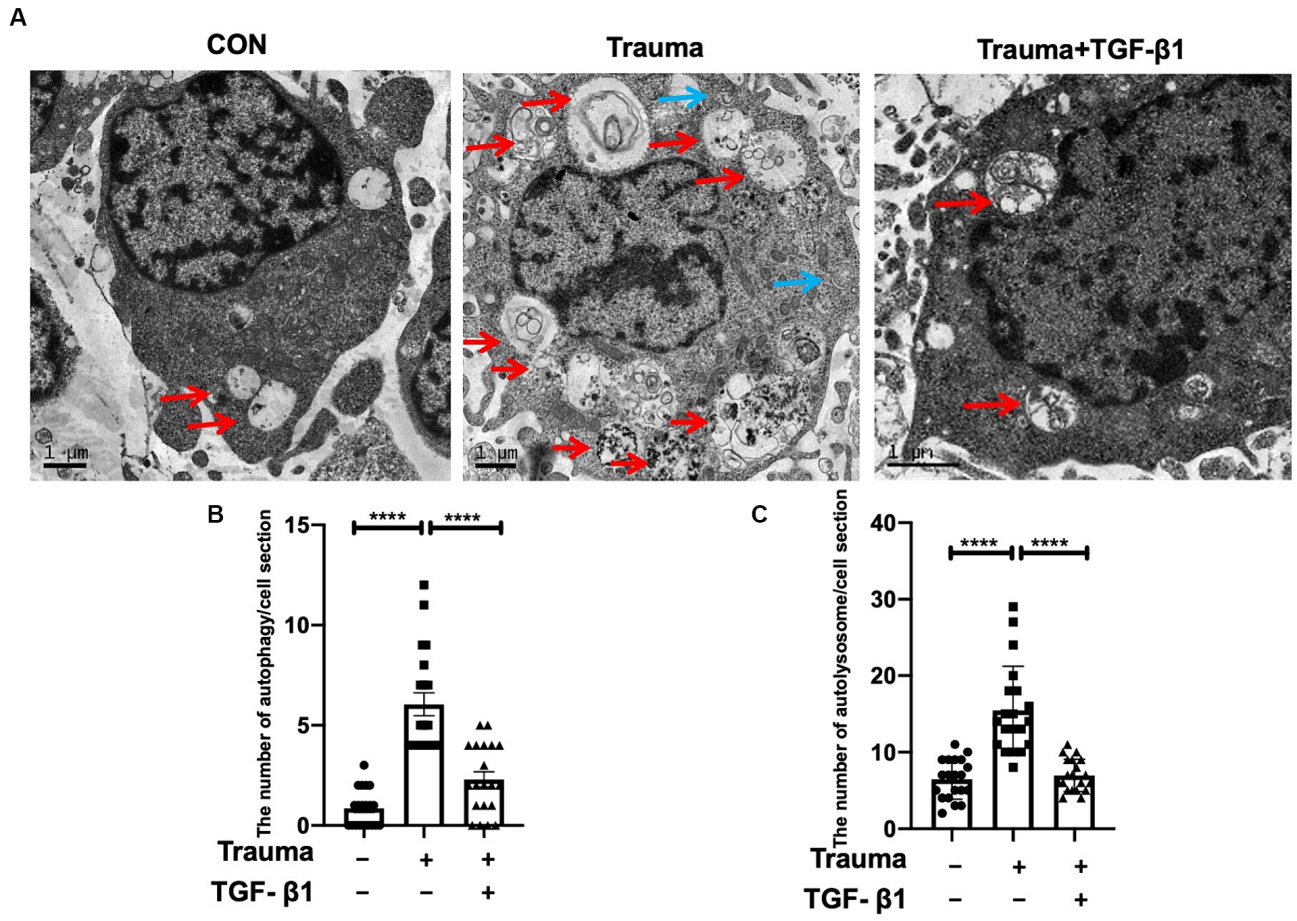
Transmission electron microscopy revealed the presence of autophagosomes and autophagolysosomes. **(A)** Representative TEM images of autophagosomes (blue) and autolysosomes (red) within neuronal cytoplasm. Scale bar: 1 μm. **(B)** Quantitative analysis of autophagosomes in the specified groups. **(C)** Quantitative analysis of autolysosomes in the specified groups. Statistical comparisons were performed using one-way ANOVA. Data are presented as mean ± SEM, ^*^p < 0.05, ^**^p < 0.01, ^***^p < 0.001, ^***^p < 0.0001.

In terms of understanding the mechanism of cell demise in mechanically injured cortical neurons, our analysis of transcriptomic data indicates that apoptosis and autophagy are the primary processes involved. Gene expression levels relevant to these processes are increased, while those related to pyroptosis are minimal. Therefore, the data suggested a relatively high prevalence of genes associated with apoptosis and autophagy. Comparing the Trauma group’s neuron cytoplasm to the normal group, we observed a higher number of autophagosomes and autophagolysosomes by using TEM. Additionally, TGF-β1 treatment reduced the count of both autophagosomes and autophagic lysosomes with statistical significance (Figures 4B,C).

### 3.4 TGF-β1 inhibits autophagy and lysosomomes in trauma-injured cortical neurons

**Figure 5:**
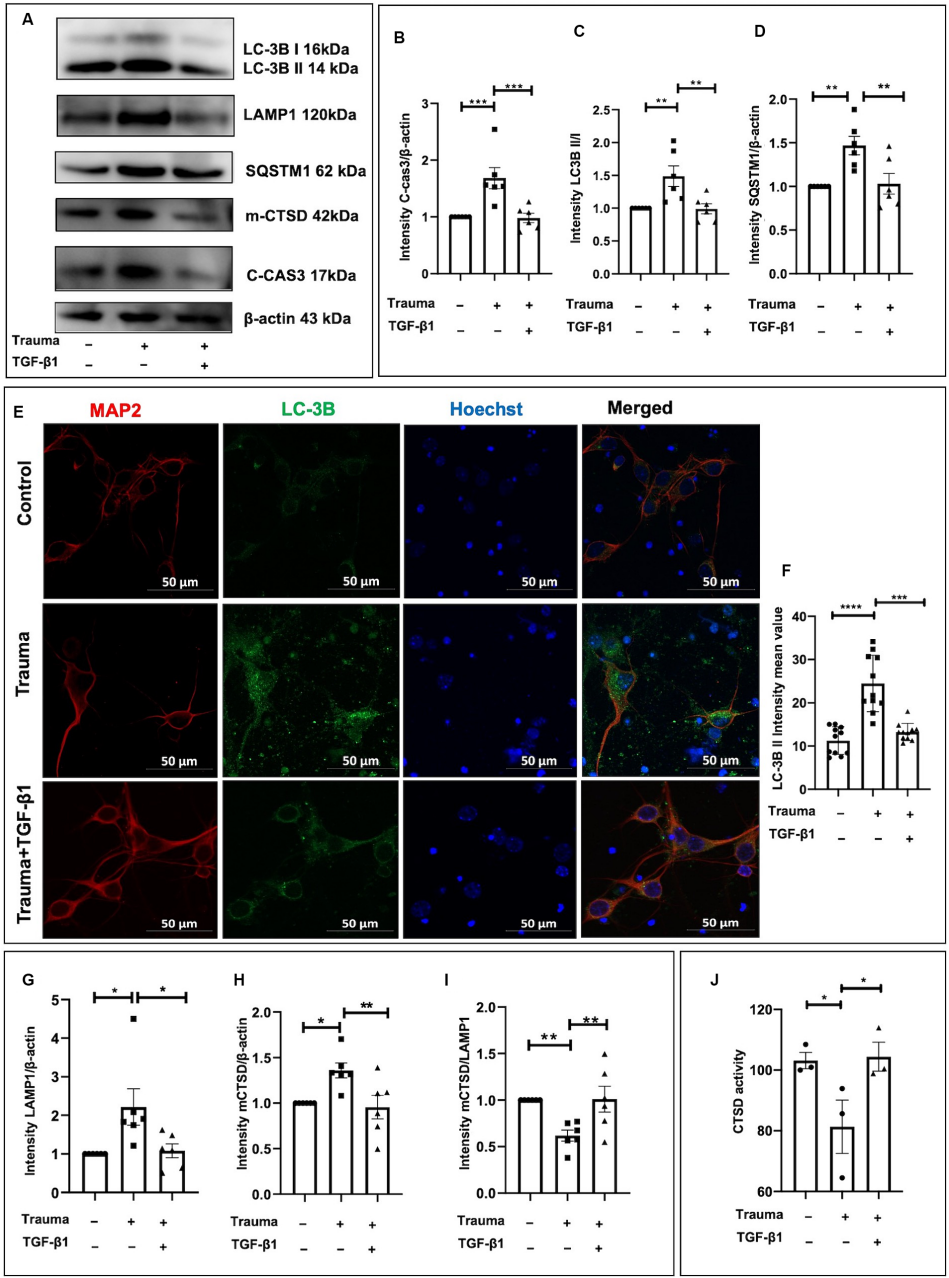
TGF-β1 alleviates the upregulation of autophagy and lysosomes induced by trauma in murine cortical neurons. **(A)** Representative western blot images showing C-cas3 expression, LC3-II to LC3-I ratio, SQSTM1, LAMP1, proCTSD, as well as mCTSD under TGF-β1 treatment conditions. **(B–D)** Statistical analysis of primary cortical neurons after 24 h of trauma injury, evaluating C-cas3, LC3-II:LC3-I ratio, SQSTM1, LAMP1, mCTSD levels with β-actin as the loading control. **(E,F)** Fluorescence microscopy to observe the intracellular expression and distribution of autophagosomes, specifically evaluating LC3B expression. **(G–I)** Statistical analysis of primary cortical neurons following 24 h of trauma injury, evaluating LAMP1, mCTSD levels with β-actin as the loading control. **(J)** Assessment of CTSD enzymatic activity in the specified groups using a Fluorometric Assay Kit. Group comparisons were conducted using one-way ANOVA, and data are presented as mean ± SEM (*n* = 6). ^*^p < 0.05, ^**^p < 0.01, ^***^p < 0.001, ^****^p < 0.0001.

TGF-β1 plays a protective role in trauma-injured neurons, with apoptosis and autophagy being associated with its effects. Our previous research has demonstrated the inhibitory effect of TGF-β1 on apoptosis in trauma-injured cortical neurons. In accordance with these results, we observed an increase in expression of the neuron apoptosis-related protein Cleaved-caspase3 at 24 h post-injury with statistical significance. TGF-β1 inhibits neuronal apoptosis induced by mechanical damage (Figures 5A,B). To investigate autophagy in cortical neurons following trauma, we assessed autophagy levels by evaluating the conversion of light chain 3 (LC3)-I to LC3-II as well as the protein levels of SQSTM1/p62 through western blot analysis at 24 h after injury. The ratio of LC3-II to LC3-I increased at 24 h after traumatic injury, indicating enhanced autophagy (Figures 5A,C). Additionally, the protein level of SQSTM1/p62 was significantly elevated after 24 h of trauma, suggesting disrupted autophagic flux (Figures 5A,D). We also examined the impact of TGF-β1 treatment, conversely, TGF-β1 treatment for 24 h reduced SQSTM1/ P62 expression back to control levels. To further visualize neuronal autophagy after mechanical injury, we labeled neurons using the neuronal marker MAP2 and autophagosomes using LC-3B (Figure 5E). The fluorescence intensity of LC-3B, indicating the number of autophagosomes, was significantly higher in mechanically injured cortical neurons. After 24 h of TGF-β1 treatment, LC-3B expression levels decreased, in accordance with the results obtained from western blot experiment (Figures 5E,F). As lysosomes play a crucial role in autophagy, we analyzed the protein levels of the lysosomal marker LAMP1 to assess the downstream processes of autophagy. We observed an increased level of LAMP1-expressing protein in cortical neurons 24 h after mechanical injury, suggesting lysosomal accumulation (Figures 5A,G). To summarize, our experimental findings demonstrate over-activation of autophagy in neurons 24 h following mechanical injury, accompanied by autophagy dysfunction, disrupted autophagic flux, and increased LAMP1 accumulation, TGF-β1 significantly reversed these changes.

### 3.5 Mechanically injured neuron’s lysosomal function impairment due to dysfunction of lysosomal enzymes

To investigate whether the degradation process within lysosomes was compromised in mechanically injured neurons. We assessed the expression levels of lysosomal proteases involved in the breakdown of autophagic lysosomal cargoes, including CTSB, CTSF, cathepsin L (CTSL), and CTSD. Previous studies have demonstrated that CTSD deficiency or double deficiency of CTSB and cathepsin L strongly inhibited autophagic flux (Marques et al., 2020). Initially, we analyzed the protein levels of mCTSD. Using β-actin as a loading control, we found a substantial upregulation of mCTSD relative expression compared to the control after 24 h of mechanical injury to cortical neurons. This indicates increased expression of lysosomal enzymes and enhanced lysosomal degradation capacity (Figures 5A,H). However, when normalizing mCTSD protein levels to LAMP1 expression, we observed a significantly lower expression of mCTSD/LAMP1 in the mechanical injury group than in the normal group at 24 h post-injury (Figures 5A,I). This suggested asynchronous expression of mCTSD and LAMP1 after mechanical injury, as they should align under physiological conditions. These findings indicated that despite increased expression levels of lysosomal enzymes, the lysosomal degradation process remained restricted. Mechanically damaged cortical neurons exhibited notable impairment of lysosomes, suggesting significant lysosome-associated dysfunction. To explore the underlying mechanisms contributing to lysosome-associated issues following upregulated autophagy, we analyzed the comparative enzymatic activities of CTSD and the accumulation of lysosomes under different experimental conditions. Notably, there was an increase in CTSD activity in the TGF-β1 treatment group, while the Trauma group showed a decrease compared to the controls, indicating impaired lysosomal enzyme activity leading to lysosomal dysfunction (Figure 5J). The diminished activity of obstructed lysosomal enzymes leads to lysosomal dysfunction, which can be reversed by TGF-β1 treatment. TGF-β1 (10 ng/mL) have a neuroprotective effect against traumatic brain injury by promoting autophagic flux, enhancing mCTSD activity, inhibiting apoptosis, and maintaining calcium homeostasis in mechanically injured neurons.

## 4 Discussion

TBI is a mechanical injury that immediately harms the impact site through direct trauma, resulting in focal cortical injury and diffuse axonal injury. Currently, surgical treatment is the primary approach for severe TBI in clinical practice, but progress in drug-based therapies has been limited despite their potential to prevent secondary injury-related cell death and lesion spread (Kalra et al., 2022). Improving the prognosis and quality of life for individuals with TBI is of utmost importance. Mechanically damaged neurons in TBI patients often experience a loss of biological function, leading to cognitive impairment. It is crucial to identify a medication that can prevent the death of damaged neurons while preserving their normal activity (Dai et al., 2023; Yang et al., 2024). TGF-β1, a cytokine involved in regulating various cellular pathways, has shown multiple benefits in TBI treatment by contributing to immune regulation in traumatic brain injuries. It improves the immune microenvironment and suppresses inflammatory responses, thereby preventing secondary craniocerebral harm in TBI patients (Zhao et al., 2020). Not only does TGF-β1 regulate the TBI immune microenvironment, providing anti-inflammatory effects and preventing secondary damage, but it also directly protects mechanically damaged neurons (Li et al., 2022). Therefore, TGF-β1 surpasses other treatments as the preferred option for TBI.

The objective of our study was to investigate the protective effect of TGF-β1 on mechanically injured neurons using an *in vitro* neuronal scratch cell model and explore its molecular mechanism. This cellular model, which is based on mechanical injury to primary cultured neurons, has been used in numerous studies and provides an essential tool for the treatment of TBI (Ni et al., 2019; Beltrán et al., 2023; Wang et al., 2024), that can reproduce neurological injuries such as stab wounds, penetrating skull frcatures, and gunshot that result in transection of the cell body (Morrison et al., 1998). We found that treatment with TGF-β1 (10 ng/mL) for 24 h significantly inhibited LDH release induced by mechanically injured cortical neurons and reduced cytotoxicity in our *in vitro* model, consistent with our previous findings (Li et al., 2022). Our pre-test dose–response analysis determined that a dose of 10 ng/mL of TGF-β1 is neuroprotective. Previous studies have also shown that TGF-β1 (10 ng/mL) has a protective effect in an *in vitro* cellular model (Ni et al., 2019), additionally, Xie et al., demonstrated that TGF-β1 (10 ng/mL) has an anti-inflammatory effect on microglia activation-induced sepsis (Xie et al., 2022).

Following traumatic brain injury, neurons experience an influx of calcium ions. In our simplified *in vitro* model, we demonstrated that intracellular Ca^2+^ concentration in neurons remains elevated after mechanical injury. Additionally, the addition of TGF-β1 could maintain intracellular calcium ion homeostasis in mechanically injured neurons. Neurons, which are long-lived and highly polarized cells, rely on autophagy to maintain cellular homeostasis (Stavoe and Holzbaur, 2019). Our research has shown that mechanically injured neurons trigger programmed cell death pathways and exhibit high expression levels of genes linked to autophagy, apoptosis. Thus, we concluded that TGF-β1 likely provided neuronal protection through apoptotic and autophagic death pathways. TBI is a significant health issue that can result in autophagy and apoptosis (Yang et al., 2024). TGF-β1 prevents neuronal calcium overload and significantly increases the expression of the anti-apoptotic Bcl2 protein in neurons (Prehn et al., 1993). However, there have been limited reports in the literature regarding the mechanism underlying the regulatory effect of TGF-β1 on autophagy in mechanically injured neurons.

TGF-β1 is a multifunctional cytokine that depend on both classical Smad-dependent and non-classical Smad transduction pathways. Its downstream cascades are mediated by specific environmental and cellular signals, such as TGF-β1 binds to its receptor TGFβR2, forming heteromeric complexes with two different type 1 receptors, ALK1 or ALK5, which activate distinct downstream pathways, often with opposing functions (Abe et al., 1996; Knöferle et al., 2010; Li et al., 2015). Endogenous TGF-β1 plays a crucial role in the functional recovery after brain injury by activating downstream Smad3 signalling (Wang et al., 2015). The TGF-β-Smad3 signalling pathway in neurons and astrocytes differentially regulates dendritic growth and synaptogenesis (Yu et al., 2014), and Kaiser et al. (2020) demonstrated *in vitro*-based experiments that TGF-β1 induces axon growth through ALK5/PKA/SMURF1-mediated RhoA degradation and PAR6 stabilisation. TGF-β1 can also alleviate sepsis by regulating the microglia NF-κB/ERK1/2 signalling pathway (Xie et al., 2022). Furthermore, our previous study reported that TGF-β1 upregulated C_av_1.2 channels in the p38 MAPK pathway, leading to increased cortical neuronal activity and inhibition of apoptosis in murine models of trauma-induced injury (Li et al., 2022). Additionally, TGF-β1 decreased the increase in Ca^2+^ concentration induced by KCl among mouse cortical neurons caused by LTCCs through the MEK, JNK1/2, and p38 MAPK pathways (Liu et al., 2018). In our transcriptome results, our results indicate that TGF- β 1 also regulates signaling pathways such as Apelin, wnt, P13K–akt, Erbb, Hif-1a pathways in trauma-injured neurons. In trauma-injured neurons, our results also demonstrated that TGF-β1 (10 ng/mL) had a significant impact on the transcriptome expression of damaged neurons after 24 h of treatment, primarily regulating synaptic homeostasis, autophagy, and apoptosis-related DEGs.

Autophagy is crucial in maintaining neuronal homeostasis / functions and has been widely recognized as a vital therapeutic target for safeguarding against injury-related damage (Bingol and Sheng, 2011; Khan et al., 2017; Marques et al., 2020). Treatment with TGF-β1 (10 ng/mL) for 24 h resulted in a big reduction in protein expression of LC-3BII/I, LAMP1, mCTSD, SQSTM/P62, and Cleaved-Caspase3. Moreover, it significantly upregulated the enzymatic activity of the CTSD in mechanically injured neurons. CTSD is a crucial lysosomal protease required for maintaining cellular protein homeostasis by breaking down endocytosis, phagocytosis, and autophagic cargo. Impairment of the autophagosome-lysosome mechanism occurs when there is CTSD deficiency or dysfunction under normal conditions (Marques et al., 2020). The elevated lysosomes and reduced activity of lysosomal enzymes in cortical neurons following mechanical injury for 24 h potentially contribute to impaired autophagic flux. Enhanced autophagy was observed in peripheral neurons at the injury site in TBI mice, which is consistent with the up regulation of autophagy in trauma cortical neurons *in vitro* (unpublished data). Although our constructed *in vitro* model cannot fully mimic the *in vivo* environment, it can still reflect the *in vivo* situation to some extent. In contrast, treatment with TGF-β1 for 24 h significantly improved this phenomenon. TGF-β1 inhibited apoptosis, upregulated lysosomal enzyme activity, reduced lysosomal dysfunction, and decreased the accumulation of autophagic lysosomes. It promoted complete autophagic flux, inhibited lysosomal proliferation, reduced the accumulation of autophagy and autolysosome, and enhanced autophagic flux, playing a neuroprotective role in mechanically injured neurons at 24 h. Apoptosis along with autophagy represent the primary modes of programmed cell death, with some studies suggesting that autophagy inhibited or delayed apoptosis (Zhang et al., 2018; Wu et al., 2020; Zheng et al., 2020), while others indicate that autophagy promoted apoptosis (Yang et al., 2020). Although autophagy and apoptosis are distinct cellular processes with different biochemical and morphological characteristics, the protein networks that regulate and execute them are highly interconnected. In our study, TGF-β1 exhibited co-inhibition of autophagy and apoptosis in mechanically injured cortical neurons, potentially blocking apoptosis through autophagy. We have demonstrated the protective role of TGF-β1 in mechanically injured neurons *in vitro*. Mechanically injured neurons undergo axonal damage and calcium overload, which leads to the upregulation of autophagy (Høyer-Hansen et al., 2007), in our study, injured neurons with autophagy dysfunction also upregulated the expression of C-cas3, promoting apoptotic pathways. This supports the role of impaired autophagy in mediating apoptotic neuronal death. In annulus fibrosus cells, TGF-β1 reduces oxidative stress-induced autophagy and apoptosis through the ERK signaling pathway (Ni et al., 2019). TGF-β1 may alleviate calcium overload, inhibit apoptosis, and attenuate autophagy in injured neurons through similar signalling pathways. These pathways include TGF-β-activated kinase 1 (TAK1), phosphatidylinositol 3-kinase (PI3K), MEK, JNK1/2, and p38 MAPK pathway. A clearer understanding of the downstream signalling pathways through which TGF-β1 regulates autophagy and apoptosis to exert protective effects on injured neurons may offer new clinical targets for future therapeutic intervention.

Although the mechanism of action of TGF-β1 is complex, many studies have elucidated its protective effects in TBI patients or animal models (Morganti-Kossmann et al., 1999). TGF-β1 may act as a bi-directional cytokine in various neurological diseases, depending on its concentration and distribution. It should be noted that the protective effect of TGF-β1 is not dose-dependent, and excessive doses can result in adverse effects. Further research is required to investigate the impact of TGF-β1 on drug efficacy, considering dosage, administration route, and timing, as these factors may influence its effectiveness. Additionally, the heterogeneity of population genetics and pathology levels should be considered when developing new anti-inflammatory drugs. Our study did not investigate the long-lasting protective effects of TGF-β1. Extensive further investigation is necessary to gain a better understanding of the protective function of TGF-β1-controlled autophagy and apoptosis in neurons that have suffered mechanical damage. Considering the role of TGF-β1 in modulating the immune microenvironment in TBI, reducing inflammation, and directly protecting mechanically injured neurons, utilizing TGF-β1 as a therapeutic agent for TBI holds great promise. Our study demonstrated that TGF-β1 (10 ng/mL) might have a protective effect on TBI by promoting autophagic flux, enhancing mCTSD activity, inhibiting apoptosis, and maintaining calcium homeostasis in mechanically injured neurons, further supporting the existing evidence that highlights the significance of TGF-β1 as a pharmacological intervention in the treatment of TBI.

## Data availability statement

The data presented in the study are deposited in the GEO database at the NCBI repository, accession number GSE249554.

## Ethics statement

The animal study was approved by Ethical Standards for Animal Care and Use of Research at Shantou University Medical School. The study was conducted in accordance with the local legislation and institutional requirements.

## Author contributions

YaL: Data curation, Formal analysis, Investigation, Methodology, Software, Validation, Visualization, Writing – original draft, Writing – review & editing. HD: Data curation, Formal analysis, Investigation, Visualization, Writing – original draft, Writing – review & editing. HZ: Data curation, Investigation, Validation, Writing – review & editing. LY: Data curation, Investigation, Writing – review & editing. SW: Data curation, Investigation, Writing – review & editing. HW: Data curation, Investigation, Writing – review & editing. JZ: Data curation, Investigation, Writing – review & editing. XL: Funding acquisition, Resources, Writing – review & editing. XC: Data curation, Writing – review & editing. YiL: Data curation, Writing – review & editing. RL: Funding acquisition, Resources, Writing – review & editing. GW: Conceptualization, Funding acquisition, Project administration, Resources, Supervision, Writing – review & editing. KL: Conceptualization, Funding acquisition, Project administration, Resources, Supervision, Writing – review & editing.
