# Supplementary material for: Transforming growth factor-β1 protects mechanically injured cortical murine neurons by reducing trauma-induced autophagy and apoptosis
Source: Front Cell Neurosci. 2024 May 28;18:1381279. doi: 10.3389/fncel.2024.1381279 (PMC11165077; doi:10.3389/fncel.2024.1381279)
Supplement: Supplementary file 1 [file Data_Sheet_1.docx]

Supplementary Material

# Supplementary Data

**Figure S1:** Trauma *vs* Control and Traum+TGF-β1 *vs* Trauma overlapping DEGs;

**Figure S2:** Top 20 significantly enriched KEGG pathways;

**Figure S3:** GSEA gene set enrichment analysis;

**Figure S4:** Transcription factor prediction analysis of differentially expressed genes in the transcriptome of TGF-β1-treated mechanically injured cortical neurons;

**Figure S5:** Key modules and hub gene analysis of the DEGs;

**Table S1.** The primers for RT-PCR;

**Table S2.** Trauma vs Trauma+TGF-β1 DEGs Gene Ontology Functional Clustering (Top 30);

**Table S3.** Trauma+TGF-β1 vs Trauma Differential Gene KEGG Clustering (Top 20);

**Table S4.** The list of Trauma+TGF-β1 and Trauma autophagic relate DEGs;

**Table S5.** The list of control and trauma lysosomes relate DEGs.

# Supplementary Figures and Tables

## Supplementary Figures


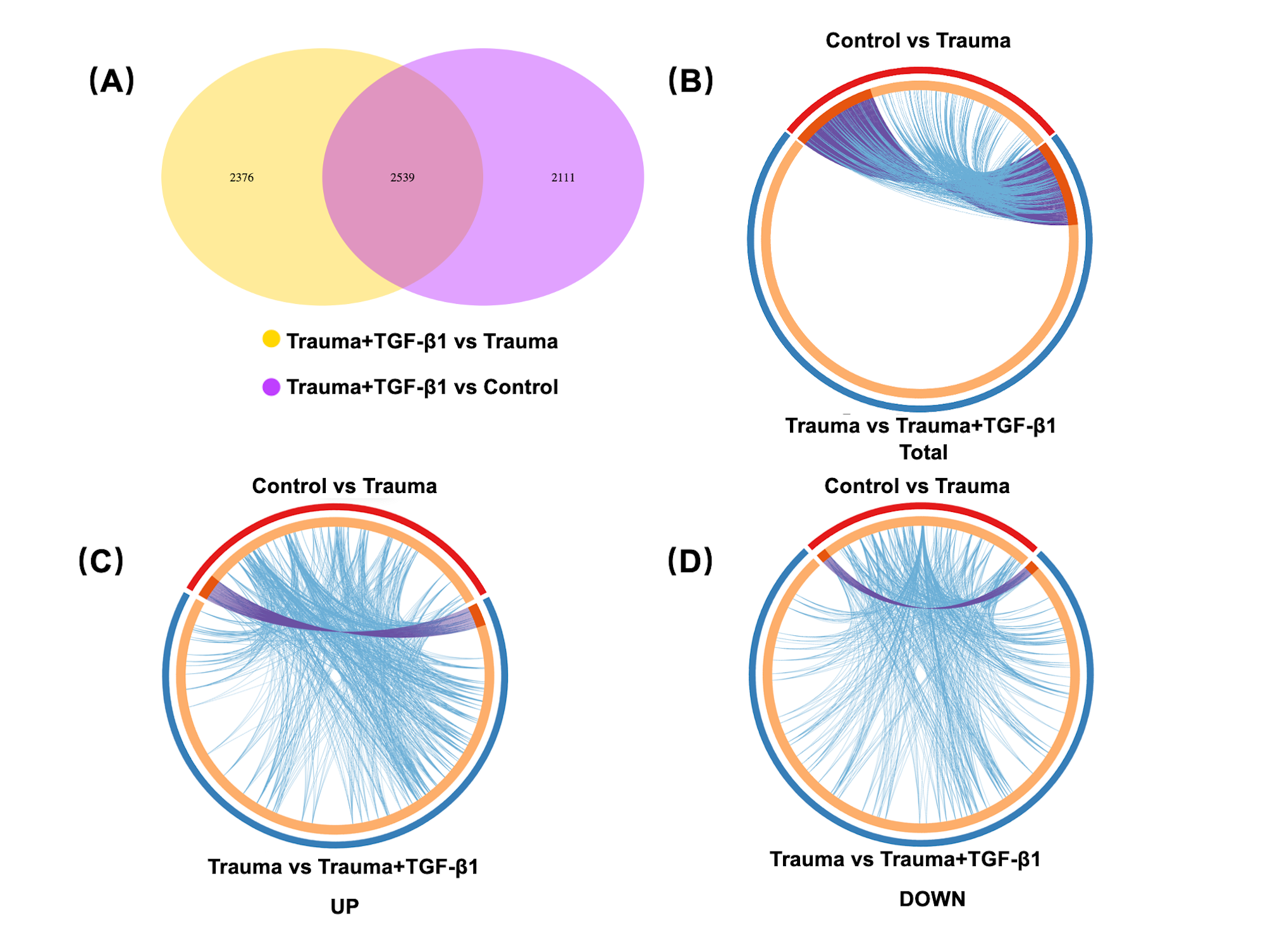


**Figure S1. Trauma** *vs* **Trauma+TGF-β1 and Trauma** *vs* **Control** **overlapping DEGs. (A)** The Venn diagram shows the number of genes differentially expressed within Trauma *vs* Control and Trauma+TGF-β1 *vs* Trauma groups. The overlapping region indicates the number of genes that are expressed in two groups. The sum of each circle is the total number of genes expressed within a group. Genes are assigned as differentially expressed at log2 (FoldChange) >=0 & *P*< =0.05, compared with the sham group. **(B-D)** The Total, up-regulate and down-regulate DEGs of Control *vs* Trauma and Trauma *vs* Trauma+TGF-β1groups Circos plot results. The Circos plot uses different colours in the outer arc to indicate significantly different sets of genes in different groups. Genes specific to the groups are represented by light orange in the inner circle, while dark orange indicates genes that overlap between groups. Purple interaction lines in the circle indicate DEGs overlap in groups, and blue interaction lines indicate the presence of functional correlation between genes in groups.


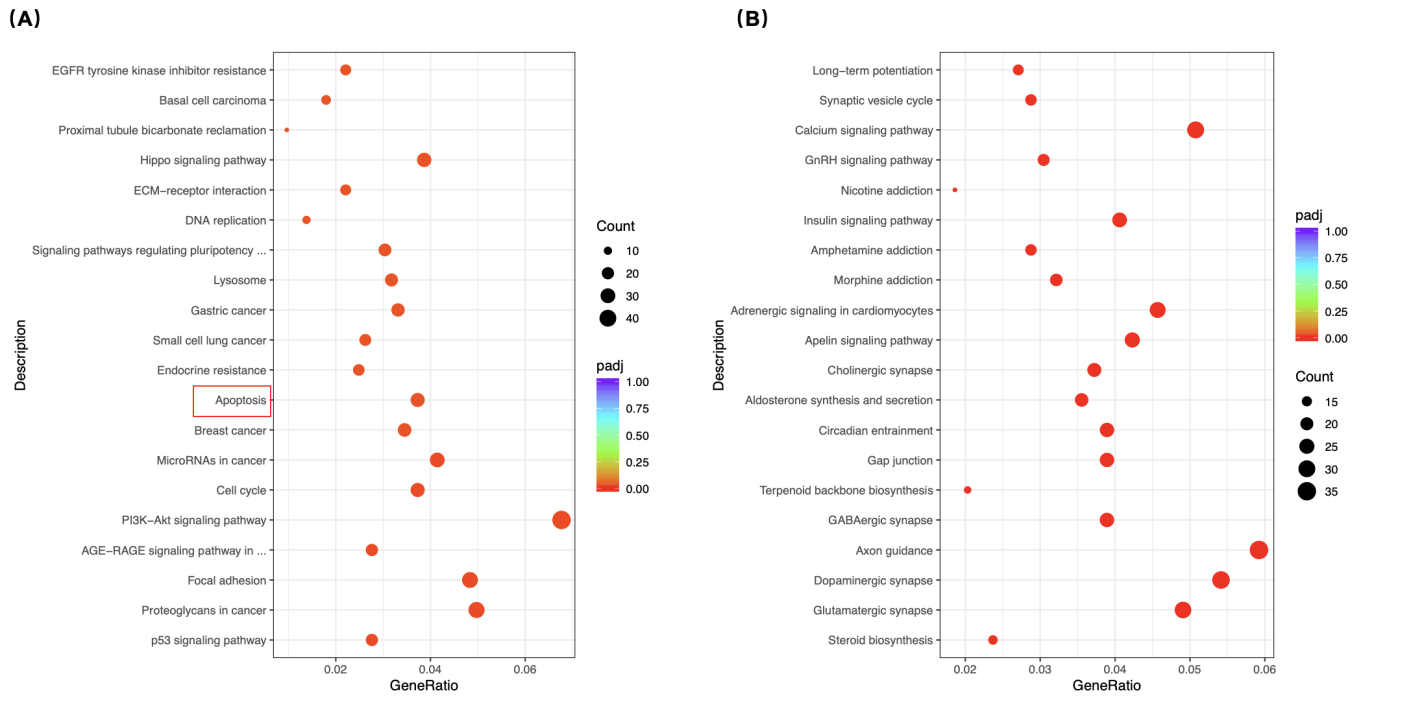


**Figure S2. Trauma VS Control group top 20 significantly enriched KEGG pathways.** Top 20 Up-regulated **(A)** and Top 20 down-regulated**(B)** significantly enriched KEGG pathways. GeneRatio represents the ratio of enriched DEGs to total DEGs. Dot size and color represent the number of DEGs enriched in the pathway and its significance, respectively.

**
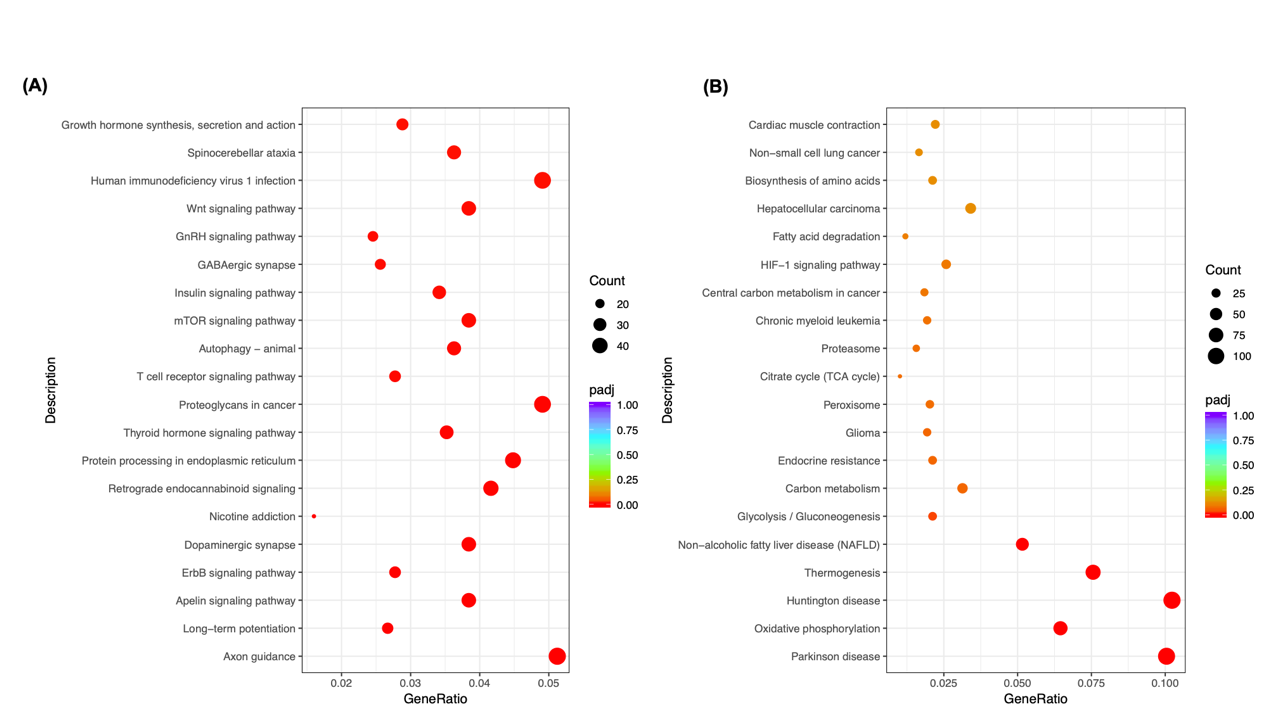
**

**Figure S3. Trauma+TGF-β1  *vs* Trauma group top 20 significantly enriched KEGG pathways.** Top 20 Up-regulated **(A)** and Top 20 down-regulated **(B)** significantly enriched KEGG pathways. GeneRatio represents the ratio of enriched DEGs to total DEGs. Dot size and color represent the number of DEGs enriched in the pathway and its significance, respectively.


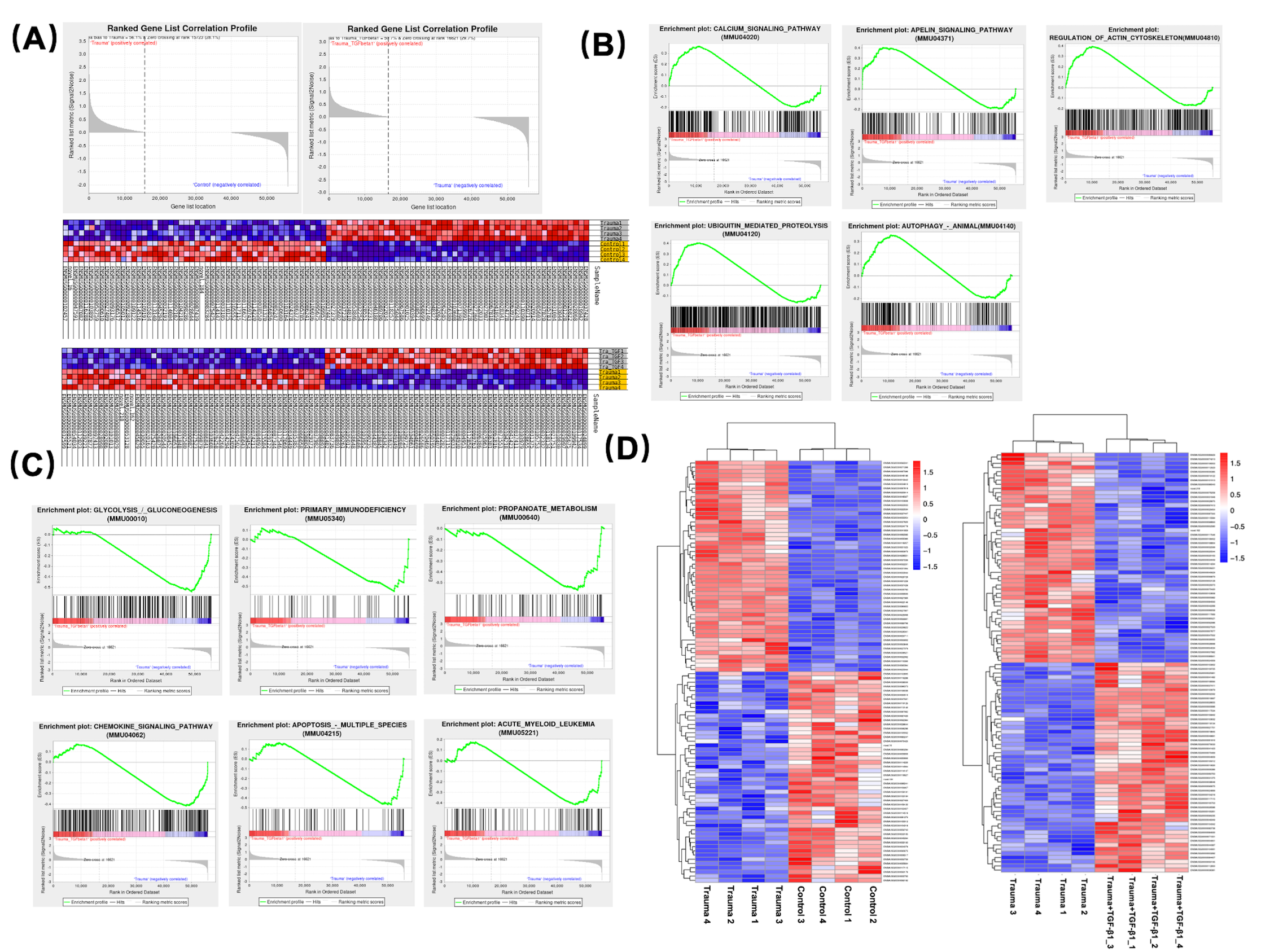


**Figure S4. GSEA gene set enrichment analysis. (A)** Differential gene-phenotype correlation analysis between the Control, Trauma, and Trauma+TGF-β1 groups. The heatmap displays the top 100 genes in different colours to indicate the strength of expression correlation, with red indicating strong correlation and blue indicating weak correlation. **(B-C)** Gene sets that were significantly up- or down-regulated after TGF-β1 treatment and had a *p*-value of <=0.05 were enriched in the KEGG signalling pathway. **(D)** The differential genes expression levels GSEA enriched in the Trauma *vs* Control and Trauma+TGF-β1 *vs* Trauma groups were analysed using a heatmap clustering analysis.


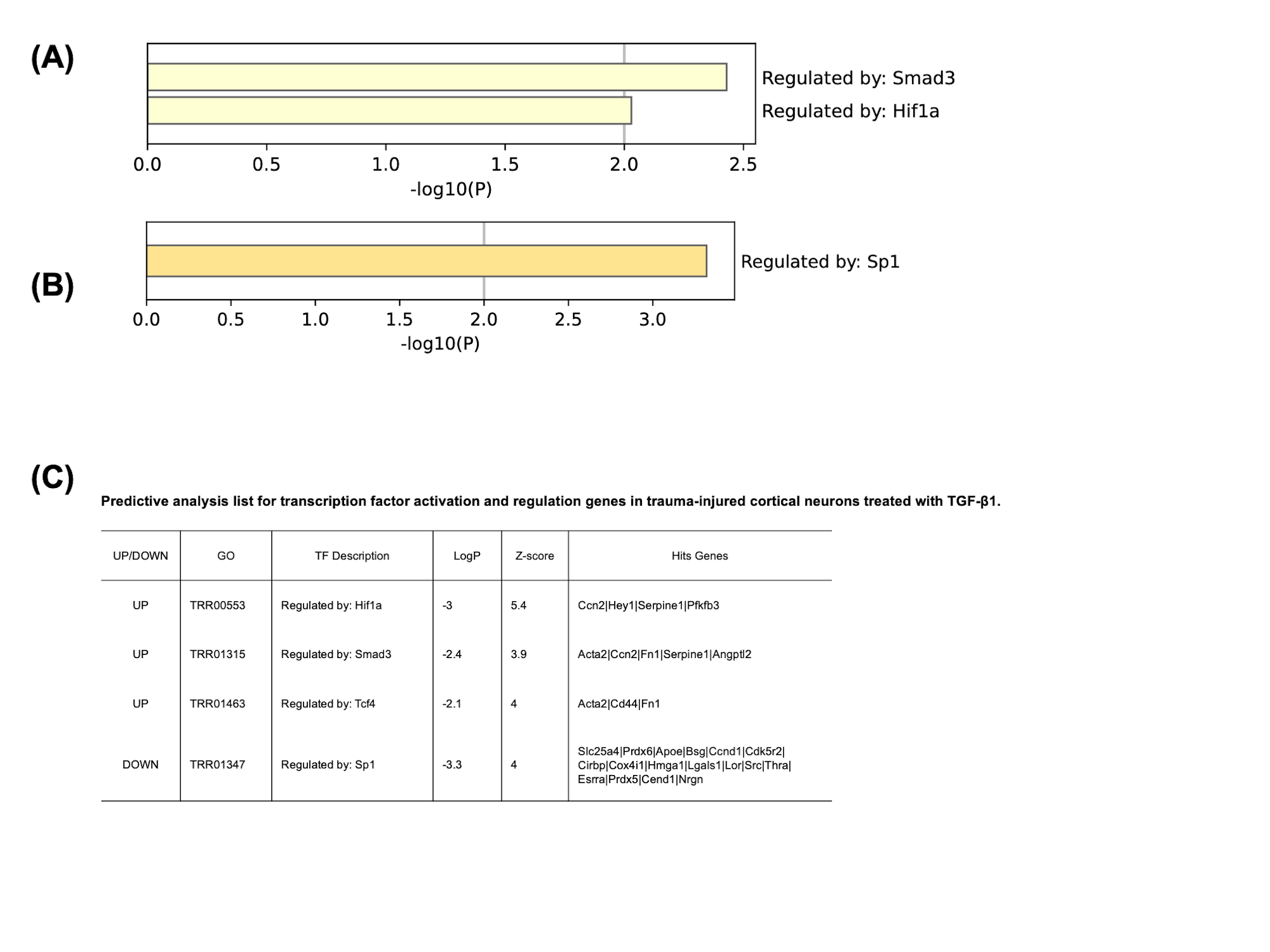


**Figure S5. Transcription factor prediction analysis of differentially expressed genes in the transcriptome of TGF-β1-treated mechanically injured cortical neurons.（A-B）**Predictive analysis of transcription factor prediction for up- and down-regulated DEGs in the transcriptome of mechanically injured cortical neurons treated with TGF-β1.**（C）**List of transcription factors and regulatory genes of DEGs in the transcriptome of TGF-β1-treated mechanically injured cortical neurons.

**
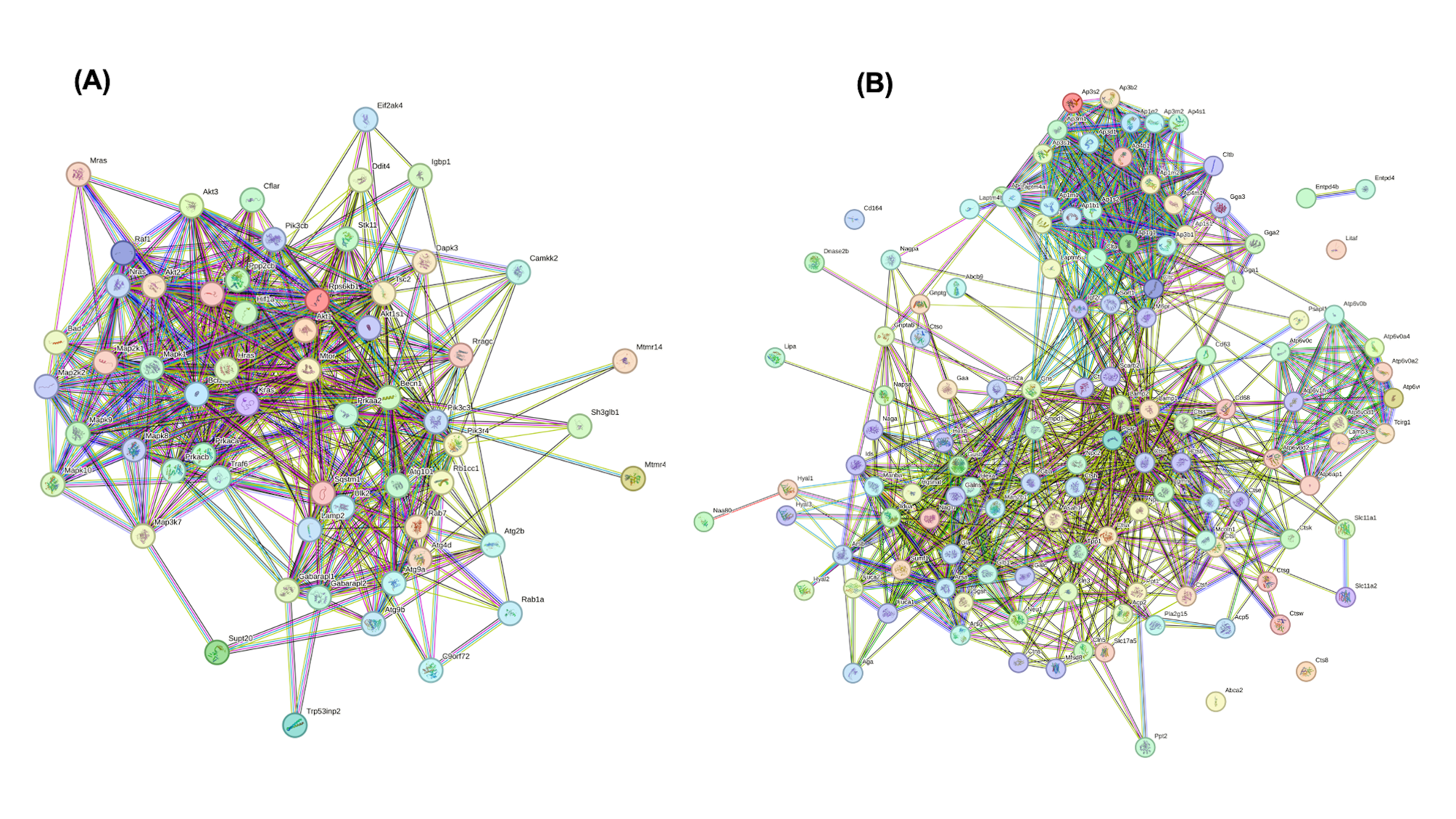
**

**Figure S6. Key modules and hub gene analysis of the DEGs. (A-B)﻿** PPI network analysis of the 59 autophagy-related genes and 150 DEGs associated with lysosomes based on the String database.

## Supplementary Tables

**Table S1.** The primers for RT-PCR.

| Gene | 5’ primer sequence | 3’ primer sequence |
| --- | --- | --- |
| β-actin | CAGCCTTCCTTCTTGGGTATG | GGCATAGAGGTCTTTACGGATG |
| Beclin1 | ATGGAGGGGTCTAAGGCGTC | TCCTCTCCTGAGTTAGCCTCT |
| SQSTM/P62 | AGGATGGGGACTTGGTTGC | TCACAGATCACATTGGGGTGC |
| lamp1 | CAGCACTCTTTGAGGTGAAAAAC | ACGATCTGAGAACCATTCGCA |
| CTSD | GCTTCCGGTCTTTGACAACCT | CACCAAGCATTAGTTCTCCTCC |
| CTSB | TCCTTGATCCTTCTTTCTTGCC | ACAGTGCCACACAGCTTCTTC |

**Table S2.** Trauma+TGF-β1 *Vs* Trauma DEGs Gene Ontology Functional Clustering (Top 30).

| Category | GOID | Description | GeneRatio | BgRatio | pvalue | padj | Count | Up | Down |
| --- | --- | --- | --- | --- | --- | --- | --- | --- | --- |
| CC | GO:0098978 | glutamatergic synapse | 221/4252 | 463/16376 | 1.61E-24 | 1.25E-21 | 221 | 112 | 109 |
| CC | GO:0099572 | postsynaptic specialization | 198/4252 | 415/16376 | 4.76E-22 | 1.84E-19 | 198 | 98 | 100 |
| CC | GO:0098984 | neuron to neuron synapse | 191/4252 | 409/16376 | 5.30E-20 | 1.37E-17 | 191 | 92 | 99 |
| CC | GO:0014069 | postsynaptic density | 181/4252 | 383/16376 | 1.06E-19 | 2.06E-17 | 181 | 86 | 95 |
| CC | GO:0032279 | asymmetric synapse | 182/4252 | 387/16376 | 1.61E-19 | 2.50E-17 | 182 | 87 | 95 |
| CC | GO:0098798 | mitochondrial protein complex | 128/4252 | 254/16376 | 4.56E-17 | 5.89E-15 | 128 | 15 | 113 |
| CC | GO:0097060 | synaptic membrane | 198/4252 | 473/16376 | 1.77E-14 | 1.80E-12 | 198 | 104 | 94 |
| CC | GO:0005840 | ribosome | 112/4252 | 226/16376 | 1.86E-14 | 1.80E-12 | 112 | 19 | 93 |
| CC | GO:0070469 | respiratory chain | 56/4252 | 86/16376 | 2.56E-14 | 2.18E-12 | 56 | 10 | 46 |
| CC | GO:0044391 | ribosomal subunit | 97/4252 | 187/16376 | 2.82E-14 | 2.18E-12 | 97 | 12 | 85 |
| MF | GO:0003735 | structural constituent of ribosome | 88/4224 | 164/16374 | 2.23E-14 | 2.45E-11 | 88 | 8 | 80 |
| CC | GO:0098800 | inner mitochondrial membrane protein complex | 69/4252 | 125/16376 | 3.31E-12 | 2.33E-10 | 69 | 7 | 62 |
| BP | GO:0099177 | regulation of trans-synaptic signaling | 196/4236 | 476/16318 | 1.57E-13 | 7.67E-10 | 196 | 87 | 109 |
| BP | GO:0050804 | modulation of chemical synaptic transmission | 195/4236 | 475/16318 | 2.55E-13 | 7.67E-10 | 195 | 87 | 108 |
| CC | GO:0098803 | respiratory chain complex | 48/4252 | 77/16376 | 1.87E-11 | 1.21E-09 | 48 | 8 | 40 |
| BP | GO:0050773 | regulation of dendrite development | 89/4236 | 174/16318 | 9.03E-13 | 1.81E-09 | 89 | 46 | 43 |
| BP | GO:0050808 | synapse organization | 180/4236 | 437/16318 | 1.52E-12 | 2.28E-09 | 180 | 85 | 95 |
| CC | GO:0005746 | mitochondrial respiratory chain | 46/4252 | 74/16376 | 5.65E-11 | 3.37E-09 | 46 | 6 | 40 |
| CC | GO:0005743 | mitochondrial inner membrane | 164/4252 | 409/16376 | 1.85E-10 | 1.02E-08 | 164 | 26 | 138 |
| BP | GO:0016358 | dendrite development | 121/4236 | 269/16318 | 8.71E-12 | 1.05E-08 | 121 | 66 | 55 |
| CC | GO:0044445 | cytosolic part | 108/4252 | 242/16376 | 2.04E-10 | 1.05E-08 | 108 | 30 | 78 |
| CC | GO:0044309 | neuron spine | 95/4252 | 206/16376 | 2.91E-10 | 1.41E-08 | 95 | 44 | 51 |
| BP | GO:0022604 | regulation of cell morphogenesis | 192/4236 | 486/16318 | 2.34E-11 | 2.08E-08 | 192 | 100 | 92 |
| BP | GO:0007005 | mitochondrion organization | 179/4236 | 446/16318 | 2.42E-11 | 2.08E-08 | 179 | 53 | 126 |
| BP | GO:0010769 | regulation of cell morphogenesis involved in  differentiation | 136/4236 | 317/16318 | 2.95E-11 | 2.22E-08 | 136 | 70 | 66 |
| BP | GO:0048813 | dendrite morphogenesis | 83/4236 | 167/16318 | 3.61E-11 | 2.41E-08 | 83 | 49 | 34 |
| CC | GO:0022626 | cytosolic ribosome | 57/4252 | 105/16376 | 5.90E-10 | 2.69E-08 | 57 | 8 | 49 |
| BP | GO:0050807 | regulation of synapse organization | 113/4236 | 252/16318 | 5.36E-11 | 3.06E-08 | 113 | 55 | 58 |
| BP | GO:0022613 | ribonucleoprotein complex biogenesis | 168/4236 | 416/16318 | 5.60E-11 | 3.06E-08 | 168 | 76 | 92 |
| BP | GO:0050803 | regulation of synapse structure or activity | 116/4236 | 262/16318 | 7.97E-11 | 3.77E-08 | 116 | 56 | 60 |

Note：Category: GO database classification, including Biological Process BP, Cellular Component CC, Molecular Function MF; GOID: GO number; Description GO number corresponds to the functional description; GeneRatio: Ratio of the number of differential genes annotated to the GO number to the total number of differential genes;; BgRatio: Background gene number annotated to the GO number to the total number of BgRatio: ratio of the number of background genes annotated to the GO number to the total number of background genes; *p*-value: p-value of significance test; padj: *p*-value corrected for multiple hypothesis testing; Count: number of differentiated genes annotated to the GO number; Up: number of differentiated genes up-regulated in relation to the Term; Down: number of differentiated genes down-regulated in relation to the Term.

**Table S3.** Trauma+TGF-β1 *vs* Trauma Differential Gene KEGG Clustering (Top 20).

| KEGGID | Description | GeneRatio | BgRatio | pvalue | padj | Count | Up | Down |
| --- | --- | --- | --- | --- | --- | --- | --- | --- |
| mmu05012 | Parkinson disease | 160/2022 | 430/9244 | 8.83E-14 | 2.06E-11 | 160 | 51 | 109 |
| mmu05016 | Huntington disease | 174/2022 | 481/9244 | 1.30E-13 | 2.06E-11 | 174 | 63 | 111 |
| mmu00190 | Oxidative phosphorylation | 95/2022 | 235/9244 | 6.66E-11 | 7.04E-09 | 95 | 25 | 70 |
| mmu04360 | Axon guidance | 84/2022 | 208/9244 | 9.12E-10 | 7.22E-08 | 84 | 48 | 36 |
| mmu04714 | Thermogenesis | 127/2022 | 361/9244 | 2.40E-09 | 1.52E-07 | 127 | 45 | 82 |
| mmu04723 | Retrograde endocannabinoid signaling | 73/2022 | 191/9244 | 1.68E-07 | 8.86E-06 | 73 | 39 | 34 |
| mmu04932 | Non-alcoholic fatty liver disease (NAFLD) | 80/2022 | 217/9244 | 2.58E-07 | 1.17E-05 | 80 | 24 | 56 |
| mmu04012 | ErbB signaling pathway | 44/2022 | 100/9244 | 5.77E-07 | 2.29E-05 | 44 | 26 | 18 |
| mmu01522 | Endocrine resistance | 45/2022 | 104/9244 | 7.67E-07 | 2.70E-05 | 45 | 22 | 23 |
| mmu05214 | Glioma | 39/2022 | 92/9244 | 7.52E-06 | 0.00023829 | 39 | 18 | 21 |
| mmu04720 | Long-term potentiation | 36/2022 | 86/9244 | 2.33E-05 | 0.00067109 | 36 | 25 | 11 |
| mmu04140 | Autophagy - animal | 59/2022 | 166/9244 | 3.36E-05 | 0.00088799 | 59 | 34 | 25 |
| mmu04371 | Apelin signaling pathway | 56/2022 | 156/9244 | 3.83E-05 | 0.00093426 | 56 | 36 | 20 |
| mmu04910 | Insulin signaling pathway | 56/2022 | 157/9244 | 4.71E-05 | 0.00106619 | 56 | 32 | 24 |
| mmu01521 | EGFR tyrosine kinase inhibitor resistance | 37/2022 | 93/9244 | 6.78E-05 | 0.00137386 | 37 | 21 | 16 |
| mmu04919 | Thyroid hormone signaling pathway | 54/2022 | 152/9244 | 7.23E-05 | 0.00137386 | 54 | 33 | 21 |
| mmu04722 | Neurotrophin signaling pathway | 52/2022 | 145/9244 | 7.37E-05 | 0.00137386 | 52 | 26 | 26 |
| mmu04260 | Cardiac muscle contraction | 45/2022 | 123/9244 | 0.00012906 | 0.00216685 | 45 | 21 | 24 |
| mmu04728 | Dopaminergic synapse | 57/2022 | 166/9244 | 0.0001347 | 0.00216685 | 57 | 36 | 21 |
| mmu05017 | Spinocerebellar ataxia | 60/2022 | 177/9244 | 0.00013671 | 0.00216685 | 60 | 34 | 26 |

Note: **KEGG ID**: KEGG pathway ID; **Description**: functional description corresponding to KEGG pathway number; **GeneRatio**: ratio of the number of differential genes annotated to the KEGG pathway number to the total number of differential genes; **BgRatio**: ratio of the number of background genes annotated to the KEGG pathway number to the total number of background genes; ***p-*value**: Significance test *p*-value; **padj**: *p*-value corrected for multiple hypothesis testing; **Count**: number of differential genes annotated to KEGG pathway number; **Up**: number of up-regulated differential genes associated with this Term; **Down**: number of down-regulated differential genes associated with this Term.

**Table S4.** The list of Trauma+TGF-β1 and Trauma autophagic relate DEGs.

| **genes** | **Trauma1- fpkm** | **Trauma2-fpkm** | **Trauma3-fpkm** | **Trauma4-fpkm** | **Trauma+TGFβ1 1-fpkm** | **Trauma+TGFβ1 2-fpkm** | **Trauma+TGFβ1 3-fpkm** | **Trauma+TGFβ1 4-fpkm** | **Trauma_TGFbeta1vsTrauma_log2FoldChange** |
| --- | --- | --- | --- | --- | --- | --- | --- | --- | --- |
| **Ddit4** | **57.46444** | **61.78931** | **51.6953** | **61.04605** | **45.15131** | **37.46397** | **39.36012** | **36.6228** | **-0.547992084** |
| **Akt1s1** | **32.41325** | **34.57192** | **32.05542** | **34.4541** | **26.11501** | **26.07549** | **27.11193** | **26.67632** | **-0.331772779** |
| **Sqstm1** | **127.1222** | **123.5281** | **120.944** | **121.8995** | **109.1377** | **106.4169** | **109.32261** | **109.54979** | **-0.182536646** |
| **Ppp2cb** | **86.44633** | **84.39254** | **84.63105** | **85.97914** | **72.04162** | **70.93202** | **69.73559** | **73.43173** | **-0.25365892** |
| **Sh3glb1** | **26.05156** | **27.3642** | **27.54239** | **25.36102** | **30.93071** | **33.1787** | **30.45043** | **30.30648** | **0.233139144** |
| **Hif1a** | **21.43517** | **20.56968** | **21.26827** | **20.71639** | **23.32742** | **26.56362** | **24.54263** | **26.49534** | **0.266611305** |
| **Prkaca** | **96.09559** | **95.7172** | **85.57031** | **90.95681** | **80.55948** | **76.88428** | **79.02082** | **75.34056** | **-0.2391189** |
| **Stk11** | **17.69272** | **18.15814** | **15.79534** | **18.69159** | **14.79294** | **12.60674** | **14.38081** | **12.28776** | **-0.378525821** |
| **Mras** | **18.02141** | **19.30227** | **18.56384** | **19.41175** | **16.71321** | **14.42931** | **15.46461** | **14.84285** | **-0.292480648** |
| **Trp53inp2** | **91.28282** | **90.21928** | **95.41388** | **92.47828** | **85.58211** | **79.16221** | **82.58929** | **78.70378** | **-0.178976428** |
| **Nras** | **18.2791** | **18.30945** | **18.37651** | **18.85257** | **21.09546** | **22.91173** | **20.87357** | **22.00392** | **0.236308357** |
| **Map2k2** | **22.63914** | **22.60016** | **20.73503** | **20.94342** | **18.30526** | **18.66026** | **19.06067** | **16.97482** | **-0.250310043** |
| **Kras** | **27.7743** | **26.77636** | **26.6466** | **26.43867** | **31.32135** | **32.11946** | **29.53537** | **30.26708** | **0.196607525** |
| **Akt1** | **56.79934** | **61.16132** | **56.6585** | **57.4031** | **51.80937** | **48.60183** | **53.52395** | **50.00893** | **0.196607525** |
| **Hras** | **35.22317** | **36.91015** | **33.77349** | **34.69256** | **30.84692** | **29.09748** | **32.05999** | **29.34843** | **-0.211053891** |
| **Bad** | **8.74535** | **9.41062** | **8.38036** | **9.339** | **5.74894** | **6.56363** | **7.17377** | **7.00801** | **-0.43520148** |
| **Bcl2l1** | **23.91291** | **24.49579** | **23.35984** | **22.55255** | **20.6143** | **18.96223** | **21.59689** | **19.02723** | **-0.232572173** |
| **Rb1cc1** | **5.66101** | **6.14899** | **6.21064** | **5.82378** | **6.8377** | **8.41936** | **6.71279** | **6.89246** | **0.276585904** |
| **Akt3** | **56.86279** | **58.05339** | **60.69033** | **55.81485** | **63.43428** | **69.54844** | **61.02657** | **65.97234** | **0.169115469** |
| **Mapk8** | **23.56902** | **23.38882** | **21.82605** | **21.00253** | **25.96432** | **26.72183** | **23.84741** | **24.8995** | **0.177209196** |
| **Gabarapl1** | **103.7924** | **106.0316** | **108.8247** | **109.4038** | **87.6649** | **98.52819** | **95.5799** | **99.9448** | **-0.163877243** |
| **Rab7** | **116.3622** | **114.235** | **117.5664** | **111.7519** | **122.2212** | **128.4363** | **122.53086** | **128.03436** | **0.125450687** |
| **Raf1** | **12.98386** | **12.12452** | **12.46097** | **11.56221** | **12.70816** | **14.40817** | **14.70413** | **15.43975** | **0.222836159** |
| **Pten** | **19.72614** | **19.56993** | **18.79661** | **19.17297** | **20.88709** | **22.30963** | **20.57035** | **21.1209** | **0.137042444** |
| **Atg2b** | **5.82779** | **5.54154** | **5.60743** | **5.36783** | **6.60607** | **6.53404** | **5.92332** | **6.49404** | **0.195096911** |
| **Becn1** | **8.7248** | **8.67231** | **9.23084** | **9.11768** | **7.22949** | **7.76435** | **7.64348** | **8.1514** | **-0.213936429** |
| **Akt2** | **8.38831** | **8.34289** | **8.40774** | **8.31599** | **7.39274** | **7.00951** | **7.37345** | **7.3139** | **-0.200397611** |
| **Atg9a** | **13.08213** | **12.70244** | **13.64474** | **13.95001** | **14.99419** | **14.64725** | **15.66638** | **15.18721** | **0.181767213** |
| **Rps6kb1** | **6.84569** | **7.18922** | **6.99021** | **6.86229** | **8.06039** | **8.36382** | **7.29332** | **8.60669** | **0.214036756** |
| **Prkacb** | **46.20903** | **46.96548** | **45.31012** | **46.79662** | **50.86966** | **52.78894** | **46.94542** | **51.67396** | **0.12778775** |
| **Atg4d** | **3.39766** | **3.19557** | **3.00563** | **3.68335** | **2.79269** | **2.47899** | **2.42416** | **2.8634** | **-0.329977799** |
| **Atg101** | **17.39567** | **20.56454** | **16.29209** | **17.77454** | **13.36038** | **15.36871** | **15.43689** | **13.80648** | **-0.311699492** |
| **Prkaa2** | **10.38208** | **10.0404** | **10.95891** | **9.31069** | **12.06084** | **12.01408** | **10.85465** | **11.53765** | **0.19263573** |
| **Mapk10** | **20.27234** | **20.38616** | **19.94537** | **19.49279** | **22.02917** | **21.80266** | **21.04342** | **21.30713** | **0.106858412** |
| **Mtmr14** | **6.89597** | **6.80689** | **6.71743** | **7.55447** | **5.54734** | **5.30828** | **5.96075** | **5.96357** | **-0.294816756** |
| **Camkk2** | **32.03412** | **31.39973** | **30.88348** | **31.67944** | **28.6625** | **29.0108** | **30.94121** | **28.28246** | **-0.10674355** |
| **Pik3c3** | **9.16065** | **8.66272** | **8.90349** | **8.87324** | **10.7164** | **9.91523** | **9.86422** | **10.22379** | **0.195040729** |
| **Mtor** | **15.55071** | **16.38547** | **15.75608** | **15.46339** | **16.61241** | **16.85597** | **17.30308** | **17.57254** | **0.115275227** |
| **C9orf72** | **6.15425** | **5.80249** | **5.4436** | **5.2876** | **7.55919** | **6.65473** | **7.04709** | **5.91642** | **0.261869282** |
| **Ulk2** | **43.47932** | **42.03941** | **43.4742** | **43.62676** | **45.6711** | **48.95491** | **44.1921** | **47.27729** | **0.109697143** |
| **Rragc** | **15.73872** | **15.90276** | **14.57641** | **13.57716** | **17.64575** | **16.09271** | **16.44058** | **18.07854** | **0.192545437** |
| **Atg9b** | **1.44811** | **1.20556** | **1.4334** | **1.3675** | **0.93169** | **0.79576** | **1.03342** | **0.99723** | **-0.53498799** |
| **Dapk3** | **14.78928** | **17.33941** | **16.03927** | **16.56101** | **15.37557** | **13.94542** | **12.88433** | **13.54322** | **-0.215355834** |
| **Mapk9** | **32.4827** | **30.89644** | **32.18291** | **31.59314** | **32.52542** | **34.48813** | **33.84399** | **35.15308** | **0.098638995** |
| **Mapk1** | **90.97933** | **92.51034** | **90.7138** | **84.27513** | **99.2057** | **99.94622** | **91.42799** | **93.3586** | **0.100177755** |
| **Pik3r4** | **4.46798** | **5.00766** | **4.84816** | **4.20119** | **5.32139** | **5.64542** | **4.7395** | **5.50257** | **0.196146811** |
| **Igbp1** | **4.95505** | **4.89239** | **4.5016** | **4.75871** | **4.91491** | **5.36922** | **5.89006** | **5.98723** | **0.216181202** |
| **Eif2ak4** | **3.58689** | **3.42799** | **3.80012** | **3.39203** | **3.62722** | **4.07341** | **4.37483** | **4.3724** | **0.21339166** |
| **Tsc2** | **13.25114** | **12.60099** | **13.54075** | **13.00654** | **13.63517** | **13.60933** | **14.27121** | **14.99858** | **-0.04237857** |
| **Mtmr4** | **22.36749** | **20.51037** | **21.60954** | **20.81743** | **24.77688** | **23.29175** | **21.88263** | **22.31201** | **0.114279266** |
| **Map3k7** | **12.16172** | **10.86873** | **11.94114** | **11.31547** | **12.92983** | **12.00249** | **12.43805** | **13.25474** | **0.130801298** |
| **Lamp2** | **6.17316** | **6.58836** | **6.33005** | **6.16707** | **6.61869** | **8.2452** | **6.23555** | **7.31073** | **0.170761035** |
| **Supt20** | **5.58807** | **4.73815** | **5.95768** | **6.10001** | **6.82073** | **6.33059** | **6.0248** | **6.29002** | **0.186914621** |
| **Gabarapl2** | **37.70268** | **38.70313** | **35.51843** | **37.83582** | **35.23967** | **37.87105** | **31.5442** | **31.86214** | **-0.132683694** |
| **Rab1a** | **63.76596** | **64.01372** | **60.55203** | **62.09855** | **68.70958** | **69.4304** | **61.52601** | **66.70355** | **0.090143835** |
| **Cflar** | **0.60459** | **0.78718** | **0.66969** | **0.82524** | **0.56484** | **0.61711** | **0.59987** | **0.52754** | **-0.321893501** |
| **Traf6** | **2.08981** | **1.7886** | **2.06645** | **2.05359** | **2.23195** | **2.57698** | **2.31218** | **2.17106** | **0.21774491** |
| **Pik3cb** | **4.56366** | **4.58171** | **4.36422** | **4.23546** | **4.75519** | **5.096** | **4.85322** | **4.95837** | **0.149712918** |
| **Map2k1** | **30.0632** | **30.35623** | **29.00652** | **32.92026** | **28.88559** | **28.13912** | **26.74792** | **29.6375** | **-0.108413789** |
| **Lamp1** | **107.0353** | **108.8666** | **102.5515** | **108.0699** | **102.5135** | **112.0916** | **104.05495** | **110.11925** | **0.008928759** |
| **Tsc22d1** | **55.49745** | **53.14746** | **50.54975** | **51.36625** | **54.9718** | **54.25048** | **51.14504** | **51.9819** | **0.013437987** |

**Supplementary Table 5.** The list of control and trauma lysosomes relate DEGs.

| **Genes name** | **Control1-fpkm** | **Control2-fpkm** | **Control3-fpkm** | **Control4-fpkm** | **Trauma1-fpkm** | **Trauma2-fpkm** | **Trauma3-fpkm** | **Trauma4-fpkm** | **TraumavsControl_log2FoldChange** |
| --- | --- | --- | --- | --- | --- | --- | --- | --- | --- |
| **Atp6v0d2** | **0.143581137** | **0.0433036** | **0.070251482** | **0.167205205** | **0.335730954** | **0.633083276** | **0.416508705** | **0.331132662** | **2.032473466** |
| **Psapl1** | **0** | **0** | **0** | **0.025461389** | **0.047715608** | **0.134965006** | **0.066595623** | **0.110301745** | **3.588580287** |
| **Gm12523** | **0** | **0.098174104** | **0** | **0** | **0.30445585** | **0.095684707** | **0.188854364** | **0.281518044** | **2.771834844** |
| **Aga** | **0.644063368** | **1.266830856** | **0.712462826** | **0.978305335** | **1.911954756** | **1.555731914** | **1.705873223** | **1.283549238** | **0.840177982** |
| **Gm47210** | **0.104408666** | **0.047233967** | **0.204340589** | **0.104217863** | **0.24413528** | **0.230181282** | **0.227156153** | **0.54178091** | **1.446781942** |
| **Litaf** | **1.096685868** | **0.821189441** | **0.727785007** | **0.692040162** | **1.214378885** | **1.278363269** | **1.140891339** | **1.111985354** | **0.511447831** |
| **Gm4829** | **0.17073834** | **0.566435352** | **1.336623644** | **0.795322831** | **0.425847179** | **1.053956151** | **0.396230348** | **0.787527418** | **-0.102133181** |
| **Ctso** | **1.522414086** | **1.343028642** | **0.968354217** | **1.228369138** | **1.696842655** | **1.487978435** | **1.899013026** | **1.898163208** | **0.467652805** |
| **Ctsh** | **0.216696137** | **0.235277437** | **0.212050484** | **0.418180255** | **0.391842822** | **0.36944633** | **0.465167735** | **0.424790434** | **0.615721113** |
| **Gla** | **3.224836734** | **3.332056154** | **2.70766927** | **3.000373214** | **3.705098704** | **4.371047374** | **4.694722837** | **3.907999285** | **0.446973842** |
| **Ctss** | **2.919483118** | **4.330359966** | **3.372071137** | **3.869606171** | **5.819336539** | **4.642610691** | **3.831880087** | **5.256731016** | **0.432403209** |
| **Lamp3** | **0** | **0.035045464** | **0.056854298** | **0** | **0.108682392** | **0.034156817** | **0.067415831** | **0.050247112** | **1.489764604** |
| **Slc11a1** | **0.263135201** | **0.36505928** | **0.394824074** | **0.140082309** | **0.50863164** | **0.788953334** | **0.335860029** | **0.40962585** | **0.812095208** |
| **Ctsz** | **7.712728305** | **11.34500219** | **10.06317135** | **11.0752272** | **13.28851541** | **15.07455779** | **13.2671174** | **11.58521966** | **0.407081866** |
| **Cd68** | **7.083716841** | **7.812768617** | **5.717513085** | **6.141765092** | **9.817268105** | **7.842644353** | **9.044501442** | **8.093849062** | **0.380475044** |
| **Ap1s3** | **0.971076779** | **0.73558943** | **0.685069931** | **0.76642497** | **0.781521312** | **0.896171392** | **1.159538237** | **1.347628246** | **0.412864976** |
| **Laptm5** | **2.899697122** | **1.831874874** | **2.51851934** | **2.363472939** | **3.305880428** | **3.480063894** | **2.702666491** | **2.433580304** | **0.314822768** |
| **Cd63** | **7.572676619** | **7.905788356** | **7.738525736** | **7.558837797** | **10.23617723** | **9.199686937** | **9.078781193** | **9.327585316** | **0.300773353** |
| **Sgsh** | **1.384112619** | **1.412203571** | **1.167123172** | **1.440373972** | **1.418517791** | **1.908773328** | **1.435190485** | **1.8464922** | **0.294521185** |
| **Gm6705** | **0** | **0** | **0** | **0** | **0.532464392** | **0.083671736** | **0.082572091** | **0.082058058** | **3.523976261** |
| **Ap4s1** | **12.17573093** | **15.36100935** | **14.82345402** | **14.26464809** | **16.89493438** | **14.92109166** | **15.81941823** | **19.67589115** | **0.25222993** |
| **Ctsa** | **12.43371028** | **12.74655921** | **12.23459483** | **13.59429736** | **14.95568066** | **14.04008183** | **15.51103152** | **15.93901628** | **0.248545083** |
| **Ctsc** | **0.727382172** | **0.658128227** | **0.580381103** | **1.027644111** | **0.826856938** | **0.828937964** | **1.022554688** | **0.938765089** | **0.278703913** |
| **Hexb** | **8.593172852** | **9.040001302** | **9.576856982** | **8.169018196** | **10.78012248** | **11.30665916** | **10.17876473** | **9.437107148** | **0.239899098** |
| **Mfsd8** | **0.172600368** | **0.162413798** | **0.216192083** | **0.130936559** | **0.548876482** | **0.079147739** | **0.090124095** | **0.400048285** | **0.714145405** |
| **Cd63-ps** | **6.462299813** | **3.561980893** | **4.215838271** | **4.003752518** | **4.724226931** | **6.35379258** | **6.334930801** | **4.625261029** | **0.278838863** |
| **Ctsd** | **83.37860243** | **86.67111403** | **83.26835552** | **89.40748924** | **101.6695196** | **99.4421323** | **97.50970156** | **100.752007** | **0.224010744** |
| **Ap1s1** | **36.75524685** | **45.34879833** | **46.29736735** | **40.31851512** | **47.95692147** | **48.7361216** | **47.37507964** | **52.19478592** | **0.221360359** |
| **Dnase2b** | **0** | **0** | **0** | **0.02325291** | **0.021788417** | **0.061629182** | **0** | **0** | **1.606736143** |
| **Cd164** | **6.982989582** | **6.371011683** | **4.91767743** | **6.314035344** | **6.681536492** | **7.240078279** | **7.170353233** | **7.125715885** | **0.20248665** |
| **Hyal1** | **0.326950887** | **0.716200493** | **0.538848893** | **0.480941844** | **0.62769333** | **0.622165928** | **0.76374265** | **0.446463618** | **0.253220672** |
| **Ap1m2** | **0.082795407** | **0.168553056** | **0.040510196** | **0** | **0.135518406** | **0.073012916** | **0.090066691** | **0.125308403** | **0.527321966** |
| **Gusb** | **2.815280508** | **3.452348062** | **2.657034212** | **2.439254823** | **2.659882865** | **3.705068369** | **2.7609362** | **3.757205346** | **0.183919066** |
| **Gm2a** | **15.19738087** | **13.89293218** | **12.09759912** | **12.70159936** | **14.9065086** | **15.24885459** | **15.84335735** | **14.92752782** | **0.180562078** |
| **Gm34086** | **0** | **0** | **0.08560028** | **0.087315773** | **0.081816534** | **0** | **0.152252711** | **0.075652449** | **0.856901136** |
| **Naa80** | **2.204946173** | **2.796748018** | **2.782796182** | **2.118639445** | **2.447777415** | **2.925725244** | **2.976941166** | **2.815834995** | **0.17595801** |
| **Fuca2** | **3.396882825** | **3.417083464** | **3.355028036** | **3.190604238** | **3.877673448** | **4.270029041** | **3.479460045** | **3.366564669** | **0.169250305** |
| **Gm27177** | **0** | **0.017030492** | **0.027628587** | **0.046970475** | **0.044012225** | **0.0331973** | **0.049141514** | **0.008139266** | **0.556919396** |
| **Tpp1** | **6.091651243** | **5.944400176** | **5.763995338** | **5.95488839** | **6.556904418** | **6.348617284** | **6.988086912** | **6.661576085** | **0.164467801** |
| **Laptm4b** | **9.926904449** | **10.19551748** | **10.40326092** | **9.00492345** | **10.38255194** | **13.54506387** | **9.689651638** | **10.44144324** | **0.159625951** |
| **Naga** | **5.271179079** | **5.408261674** | **5.602417115** | **4.606999755** | **5.260418977** | **6.672309576** | **5.816413958** | **5.474835907** | **0.15631333** |
| **Ctsl** | **20.24251373** | **21.35115046** | **18.95582528** | **18.50589794** | **21.82770311** | **22.85009482** | **21.16385419** | **21.94164679** | **0.154189412** |
| **Gm26646** | **0** | **0.067322597** | **0** | **0** | **0** | **0.03280775** | **0.032376578** | **0.064350051** | **0.887334553** |
| **Slc17a5** | **3.225528271** | **3.842594523** | **3.261590174** | **3.362728554** | **3.905154825** | **3.887379172** | **3.212502819** | **4.137857305** | **0.147335902** |
| **Sumf1** | **3.015892616** | **3.402302144** | **2.801806537** | **3.867768211** | **3.784849683** | **3.652682884** | **3.720957886** | **3.252077704** | **0.141800033** |
| **Naglu** | **2.98318892** | **4.513349443** | **3.206371379** | **2.855698827** | **3.727885108** | **4.420467851** | **3.511177786** | **3.235551063** | **0.136509422** |
| **Scarb2** | **23.72269552** | **24.59852538** | **25.1266991** | **26.72441987** | **27.72048314** | **27.93258099** | **27.27749639** | **26.36537794** | **0.129176474** |
| **Ppt2** | **3.853913991** | **5.286716817** | **3.844284363** | **4.392878597** | **4.581319013** | **4.801842195** | **4.543992063** | **4.967274959** | **0.122538604** |
| **Gba** | **21.04594213** | **21.81121428** | **22.95086601** | **22.49870848** | **20.90535689** | **21.14303173** | **20.5622196** | **19.46832452** | **-0.102202406** |
| **Ctsb** | **72.14796711** | **77.41710343** | **71.57872536** | **72.41281344** | **81.82647206** | **80.39843034** | **77.36012836** | **78.34625608** | **0.11832968** |
| **Asah1** | **8.12909243** | **7.840838444** | **8.630225327** | **7.540116249** | **7.674921853** | **9.400359123** | **8.626104151** | **8.721633844** | **0.103146475** |
| **Atp6v0c-ps2** | **11.60115002** | **16.0610723** | **10.94210897** | **15.4864382** | **16.60277412** | **13.18864495** | **13.86678401** | **14.26398451** | **0.099795387** |
| **Idua** | **1.623313853** | **1.594651967** | **1.285938562** | **1.589483542** | **1.677355717** | **1.785985548** | **1.695241948** | **1.363795548** | **0.100983024** |
| **Hyal2** | **3.848741865** | **5.482396149** | **4.635360466** | **3.999316963** | **5.778065998** | **4.403501437** | **4.036453782** | **4.898938303** | **0.090676816** |
| **Lgmn** | **27.978646** | **26.15019193** | **22.61490871** | **21.3924772** | **25.90692549** | **25.65981267** | **26.29652722** | **26.20541555** | **0.087947639** |
| **Lamp1** | **105.8133042** | **99.52002515** | **97.00639785** | **100.890683** | **107.0352954** | **108.8666253** | **102.5514499** | **108.0698483** | **0.0844801** |
| **Npc1** | **2.986399684** | **3.045019437** | **3.366351862** | **3.026802783** | **3.324981433** | **3.155194111** | **3.328639526** | **3.332752081** | **0.084414173** |
| **Gnptg** | **21.70333054** | **22.27788246** | **23.4438577** | **21.51943626** | **23.11035148** | **23.72627597** | **23.4899057** | **23.71857324** | **0.083906456** |
| **Npc2** | **9.83640802** | **11.51686228** | **11.76583568** | **10.55809549** | **11.08925474** | **11.21428984** | **11.53496273** | **12.13502562** | **0.077163476** |
| **Galc** | **1.546341536** | **1.324020875** | **1.342208256** | **1.683042485** | **1.421789791** | **1.436826866** | **1.630825152** | **1.715117638** | **0.078587417** |
| **Gaa** | **28.8758287** | **31.14286606** | **31.02261187** | **30.23369347** | **34.53179459** | **32.73781318** | **30.62832423** | **29.61501548** | **0.075357606** |
| **Man2b1** | **5.085532002** | **4.506088628** | **5.523287173** | **4.680162434** | **5.317014541** | **5.270193468** | **5.207977935** | **5.007473855** | **0.075436915** |
| **Arsb** | **34.77944325** | **28.57267368** | **28.86320572** | **31.54638903** | **30.20922503** | **30.16701414** | **28.66953416** | **26.66745932** | **-0.093462605** |
| **Fuca1** | **7.18192029** | **9.459985944** | **8.658745343** | **8.456010235** | **8.368787494** | **8.082904254** | **8.546438237** | **10.10609103** | **0.059489981** |
| **Lipa** | **2.434893252** | **1.621943351** | **1.819851863** | **2.009421843** | **2.23254152** | **1.902051082** | **1.877053611** | **2.163827408** | **0.056400285** |
| **Cln3** | **1.416587741** | **1.922571959** | **2.10133067** | **1.604776611** | **1.724530064** | **1.695362189** | **2.064445102** | **1.818236791** | **0.054630242** |
| **Cltb** | **39.13282062** | **44.10471625** | **42.04355896** | **38.91471014** | **38.73656577** | **43.77520291** | **42.07283798** | **45.49434915** | **0.054281031** |
| **Entpd4b** | **0.708385773** | **0.160235118** | **0.315090455** | **0.184807933** | **0.127994134** | **0.837650041** | **0.203157615** | **0.389862161** | **0.191340961** |
| **Hexa** | **15.1089174** | **18.94410992** | **17.81210606** | **18.72162166** | **18.33436384** | **19.29647975** | **16.80420647** | **18.07949498** | **0.041612424** |
| **Gns** | **11.00678215** | **11.95806753** | **11.0806671** | **10.91108703** | **11.83988883** | **10.97498112** | **11.58554121** | **11.76345101** | **0.041227459** |
| **M6pr** | **8.806194961** | **8.447234223** | **8.342866089** | **7.96554369** | **8.061557716** | **8.384229004** | **8.233348313** | **9.664845251** | **0.037122352** |
| **Arsa** | **9.73149768** | **10.33904037** | **9.67841432** | **11.45900888** | **10.29129718** | **10.74658861** | **10.68220329** | **10.40186223** | **0.035269685** |
| **Abcb9** | **6.649131967** | **5.879578909** | **6.926730069** | **5.860929025** | **6.913592072** | **6.630996293** | **6.382272723** | **5.941114714** | **0.03447707** |
| **Ctns** | **5.539186098** | **3.711160632** | **4.688956679** | **4.381160975** | **4.607180473** | **4.462163599** | **5.170800197** | **4.458987904** | **0.034496541** |
| **Hgsnat** | **10.42270172** | **12.18829274** | **11.41161815** | **11.20846558** | **11.88203823** | **11.89657656** | **11.20969223** | **11.00384897** | **0.026510024** |
| **Ctsf** | **7.710106727** | **9.087502204** | **7.55742988** | **8.442453163** | **8.308687325** | **8.004336052** | **8.336734465** | **8.697405311** | **0.026803572** |
| **Ppt1** | **13.94000626** | **12.6607986** | **13.10450826** | **12.64315285** | **13.07127136** | **12.68296359** | **14.24053919** | **13.18802974** | **0.026739744** |
| **Atp6v0c** | **109.8220889** | **125.2935399** | **124.3640285** | **114.471727** | **118.0404901** | **125.0296566** | **115.3300789** | **121.6217293** | **0.021741139** |
| **Ap3m1** | **10.20083087** | **9.087101433** | **9.565083942** | **9.470083303** | **10.67610715** | **9.306050408** | **9.707841213** | **9.086511555** | **0.020238345** |
| **Ap3s1-ps2** | **0.561264203** | **1.625044237** | **1.977233421** | **1.792763233** | **1.15489902** | **2.078787434** | **1.367644819** | **1.456211644** | **0.027322043** |
| **Gm15956** | **0** | **0.351432002** | **1.013561887** | **0.904640113** | **0.847664927** | **0.456694337** | **0.56336536** | **0.447886609** | **0.031048015** |
| **Ap1g1** | **18.79345796** | **16.47586675** | **16.94150022** | **18.73377478** | **18.08415958** | **17.9758066** | **18.56431518** | **16.70705685** | **0.011490008** |
| **Laptm4a** | **98.64631475** | **85.90427907** | **82.11094654** | **87.4887796** | **85.13298775** | **87.37891788** | **92.10048154** | **91.36861566** | **0.011146769** |
| **Igf2r** | **8.80510654** | **8.510106402** | **8.267857773** | **8.677497452** | **8.88856478** | **8.922393294** | **8.707904719** | **7.904876258** | **0.010140537** |
| **Sort1** | **36.82071137** | **36.92776965** | **36.7371659** | **38.91337199** | **36.55894373** | **37.27869343** | **37.6780301** | **36.67856405** | **-0.008144451** |
| **Acp2** | **12.80838244** | **11.94042067** | **11.82247823** | **12.18450702** | **12.01492998** | **12.80914894** | **11.00480893** | **12.52959766** | **-0.008225843** |
| **Gm44524** | **0** | **0.216997374** | **0** | **0.23939343** | **0.22431618** | **0** | **0** | **0.207416123** | **-0.109522604** |
| **6530413G14Rik** | **0.034261674** | **0.06199925** | **0.033527153** | **0** | **0.064090337** | **0** | **0** | **0.05926175** | **-0.086346452** |
| **Clta** | **150.3536997** | **145.6762443** | **144.1622857** | **133.5716922** | **139.2421502** | **154.6553508** | **132.1348414** | **141.4300255** | **-0.012546617** |
| **Ap3m2** | **17.22171916** | **19.66274348** | **20.12743514** | **19.67057458** | **18.74068735** | **20.00013368** | **18.32479983** | **18.75729323** | **-0.013030897** |
| **Smpd1** | **34.43514827** | **36.72529364** | **36.23180011** | **31.53378928** | **34.77599887** | **35.39901169** | **32.35739677** | **34.54786849** | **-0.016226287** |
| **Gm17149** | **0** | **0** | **0** | **0.173001652** | **0** | **0** | **0.150831686** | **0** | **-0.123967245** |
| **Lamp2** | **6.967573298** | **5.823989611** | **6.438669924** | **6.486203583** | **6.173156849** | **6.588360857** | **6.33005151** | **6.167068508** | **-0.021777884** |
| **Atp6v0a2** | **5.536989019** | **6.408713849** | **7.432410436** | **6.124891226** | **6.236229927** | **6.143951145** | **6.500495823** | **6.12574502** | **-0.025084232** |
| **Neu1** | **9.784851273** | **9.892941086** | **9.4387781** | **8.811127535** | **9.249542177** | **9.457842428** | **9.197177194** | **9.245325058** | **-0.026838536** |
| **Acp5** | **0.192101659** | **0** | **0** | **0.063916867** | **0.029945658** | **0.028234059** | **0.139314981** | **0.027689542** | **-0.167671821** |
| **Ap1g2** | **1.324825791** | **1.226893785** | **2.257301976** | **2.41144389** | **1.107917516** | **1.62186725** | **1.993908076** | **2.25108669** | **-0.044066172** |
| **Cts8** | **0** | **0** | **0.050779248** | **0** | **0** | **0** | **0** | **0** | **-1.587815974** |
| **Galns** | **3.556297067** | **2.396633638** | **3.336052893** | **2.374692488** | **2.133373843** | **3.26588152** | **3.479089355** | **2.502925583** | **-0.02971193** |
| **Ap4b1** | **0.335368394** | **0.485500832** | **0.59072144** | **0.624876968** | **0.522787107** | **0.414041254** | **0.466971163** | **0.58008018** | **-0.034308854** |
| **Hyal3** | **0.033364679** | **0.181128198** | **0.391792659** | **0.166518531** | **0.249649629** | **0.205957893** | **0.203251117** | **0.057710236** | **-0.110290336** |
| **Gnptab** | **28.44816697** | **25.57968585** | **25.81662554** | **27.65732683** | **27.01074894** | **26.38269541** | **25.40140959** | **26.06497553** | **-0.032369884** |
| **Ap4m1** | **2.315599586** | **2.742318986** | **3.701457679** | **3.26737871** | **2.7327577** | **2.469650656** | **2.996376521** | **3.512455282** | **-0.034207144** |
| **Psap** | **340.5573639** | **377.9118398** | **351.9379415** | **346.2712548** | **345.6194869** | **349.593107** | **341.8036187** | **341.6832924** | **-0.035792314** |
| **Glb1** | **10.47948185** | **10.14643073** | **9.113043423** | **7.742801663** | **9.498388245** | **9.336575211** | **9.289085699** | **8.334294274** | **-0.037222469** |
| **Ctsw** | **0.03444324** | **0.405130762** | **0.067409654** | **0.275042372** | **0.225504925** | **0.212615779** | **0.089923501** | **0.178727407** | **-0.15304264** |
| **Atp6v1h** | **16.45866674** | **16.24323088** | **16.41905778** | **16.32208281** | **16.52910575** | **15.66080606** | **15.24605627** | **15.95859143** | **-0.042617459** |
| **Gga1** | **16.27880854** | **17.6340769** | **20.05163266** | **16.57645311** | **17.11475853** | **16.2278721** | **17.09626079** | **17.66166196** | **-0.047435728** |
| **Ap3s2** | **12.47055871** | **12.8069241** | **12.41862196** | **13.1289357** | **12.42559687** | **12.02598773** | **12.27024065** | **12.32712133** | **-0.047964818** |
| **Ap3m1-ps** | **1.343286376** | **0.420712715** | **0.606686884** | **0.721986227** | **0.434902355** | **0.683407861** | **0.719388008** | **0.983000738** | **-0.121651594** |
| **Cln5** | **2.206208481** | **1.950267073** | **1.761869526** | **1.999677698** | **1.565399608** | **2.191526979** | **2.36134272** | **1.425530634** | **-0.065116012** |
| **Cltc** | **101.8696707** | **84.58723084** | **83.67884173** | **91.96628589** | **90.70888131** | **86.36047456** | **86.21710593** | **83.30606613** | **-0.059838411** |
| **Ctsg** | **0.062918097** | **0** | **0.30784612** | **0** | **0** | **0.110968324** | **0.054754968** | **0.108828208** | **-0.418170627** |
| **Napsa** | **0.317641778** | **0.508475425** | **0.191281228** | **0.268282637** | **0.13711959** | **0.258564538** | **0.425277309** | **0.338103876** | **-0.146144444** |
| **Ap1m1** | **30.14972391** | **29.7725304** | **31.24119885** | **29.52411202** | **29.17293917** | **28.85558047** | **27.71247995** | **29.14646023** | **-0.067662443** |
| **Atp6v0b** | **81.37767211** | **106.8032297** | **103.4175344** | **99.16385527** | **92.14779617** | **94.00688857** | **89.62755582** | **95.01670926** | **-0.072321978** |
| **Gm8532** | **0.422957015** | **0.255124895** | **0** | **0** | **0.131864826** | **0.124327851** | **0.245387775** | **0** | **-0.434730345** |
| **Ap4e1** | **1.835213794** | **1.600538123** | **1.685653637** | **1.845086426** | **1.889995296** | **1.653433681** | **1.637469371** | **1.380892399** | **-0.083319401** |
| **Ap1b1** | **31.42859875** | **32.00713454** | **31.92239437** | **30.82148153** | **31.07546121** | **29.02178728** | **30.07809589** | **28.57547287** | **-0.084403452** |
| **Pla2g15** | **8.240245192** | **11.30064419** | **9.224573736** | **8.655824441** | **10.33000949** | **8.769425555** | **8.109768699** | **7.910037254** | **-0.08997898** |
| **Gm12485** | **0** | **0.40416693** | **0** | **0** | **0.20889926** | **0** | **0** | **0** | **-0.806537321** |
| **Nagpa** | **4.36038344** | **3.771542543** | **4.427918306** | **4.160799226** | **4.668237396** | **4.11121252** | **3.221879363** | **3.676166357** | **-0.090291198** |
| **Atp6v0d1** | **157.1392643** | **140.4069829** | **140.6103721** | **137.2772451** | **132.5981356** | **138.700719** | **135.3321107** | **132.5146099** | **-0.090442817** |
| **Ap3s1-ps1** | **7.520940316** | **8.531482245** | **10.98463012** | **7.955386848** | **9.764146258** | **7.523230714** | **5.37289036** | **9.805158402** | **-0.106102086** |
| **Ap3b1** | **11.46119273** | **11.26215416** | **10.74001197** | **10.51304564** | **11.16081504** | **9.905386864** | **9.725459948** | **10.38175161** | **-0.092230181** |
| **Ap3s1** | **30.66846801** | **27.96914339** | **26.48495514** | **26.74530542** | **26.58123356** | **24.82302196** | **25.72280676** | **27.55426798** | **-0.092283377** |
| **Ctse** | **0** | **0.037101759** | **0** | **0** | **0** | **0.018080486** | **0** | **0** | **-0.806459273** |
| **Manba** | **0.829795026** | **0.742354522** | **1.04922048** | **0.828278602** | **0.88075716** | **0.822193936** | **0.697793984** | **0.806337246** | **-0.101644491** |
| **Entpd4** | **0.016099677** | **0** | **0** | **0** | **0** | **0** | **0** | **0.006961824** | **-0.876066816** |
| **Atp6ap1** | **106.877762** | **104.9612569** | **99.18478473** | **104.6242641** | **96.99028356** | **97.20465785** | **96.17015323** | **92.15355791** | **-0.116468106** |
| **Ap3b2** | **33.063174** | **31.52029284** | **34.36306107** | **32.82568063** | **31.16913262** | **30.37836469** | **29.22927521** | **30.12124765** | **-0.120825811** |
| **Ids** | **133.0212665** | **120.6445417** | **114.5965238** | **122.5400106** | **117.2295881** | **113.5403807** | **112.8243777** | **105.0255465** | **-0.126292974** |
| **Atp6v0a1** | **85.9916686** | **88.97186912** | **87.77637644** | **90.22817246** | **81.58761993** | **80.82343844** | **80.60672132** | **78.95603522** | **-0.129222188** |
| **Ctsk** | **0.472842818** | **0.816754005** | **0.84128318** | **0.429071558** | **0.160819272** | **0.720229925** | **0.67335574** | **0.557636602** | **-0.271890103** |
| **Slc11a2** | **11.87574633** | **12.85519901** | **13.66310615** | **12.6708599** | **11.93023873** | **12.01507169** | **10.99369286** | **11.34987948** | **-0.13858526** |
| **Tcirg1** | **2.089761115** | **2.695660838** | **2.639103325** | **2.607427679** | **2.047013065** | **2.390724905** | **2.199560483** | **2.430098658** | **-0.142488805** |
| **Gm20691** | **1.756605261** | **2.119145526** | **1.145964115** | **0.584465041** | **0.91275803** | **0.516352606** | **1.69855502** | **1.181586745** | **-0.378009898** |
| **Ap3d1** | **38.13335025** | **37.30643488** | **39.25653446** | **40.62550012** | **34.97759627** | **34.81087771** | **34.70237082** | **34.44219887** | **-0.157366885** |
| **Mcoln1** | **7.424243891** | **7.487974769** | **8.797227501** | **8.226451818** | **6.847388715** | **6.531877551** | **7.194701356** | **7.856719595** | **-0.163799621** |
| **Abca2** | **31.53933602** | **30.97441212** | **33.70563864** | **34.58322923** | **28.28257201** | **29.29623581** | **29.636366** | **28.75143851** | **-0.170065501** |
| **Arsg** | **0.981738168** | **0.797934788** | **0.586184409** | **0.548104312** | **0.575836748** | **0.660312664** | **0.564749975** | **0.690749855** | **-0.222301085** |
| **Gga3** | **12.2703589** | **13.23023781** | **15.18480441** | **13.47376757** | **11.43761083** | **10.6737401** | **12.31771973** | **13.03310587** | **-0.186736546** |
| **Gga2** | **4.058407283** | **4.009109226** | **5.247457546** | **4.675241696** | **3.87052758** | **3.696236844** | **3.948736168** | **4.246372183** | **-0.186806743** |
| **Gm17833** | **0.122055201** | **0.165651516** | **0.776350337** | **0.548244672** | **0.114159032** | **0.269085161** | **0.424877988** | **0.211116505** | **-0.65038028** |
| **Gm18494** | **0.619530625** | **0.152875966** | **0** | **0.281090206** | **0.210709479** | **0.049666498** | **0.196055051** | **0.146125917** | **-0.800511016** |
| **M6pr-ps** | **1.096290994** | **0** | **0.153255442** | **0.078163397** | **0.146481168** | **0.138108769** | **0.204440538** | **0.067722614** | **-1.244943593** |
| **Ap1s2** | **9.412078503** | **8.00665678** | **6.609893319** | **8.127418999** | **6.770734275** | **6.233670735** | **7.473231559** | **6.170055257** | **-0.267483499** |
| **Gm47439** | **0.260394209** | **0.094240847** | **0.101924693** | **0.051983669** | **0** | **0.091851185** | **0** | **0** | **-2.45080435** |
| **Atp6v0a4** | **0.104555632** | **0.03547534** | **0.038367791** | **0.06522785** | **0.012223946** | **0.046101057** | **0** | **0.01130299** | **-1.800767617** |
| **Gm15396** | **0.05968264** | **0.10800053** | **0.11680626** | **0.297867858** | **0.055821565** | **0** | **0** | **0.05161595** | **-2.453278322** |

Note: Each sample readcount; log2：The ratio of gene expression levels in the treatment group to the control group was then taken as a logarithmic value with a base of 2.
